# Supplementary figures and images for: Zebrafish fast muscle contractions avoid the mammalian requirement for voltage-gated Na+ channels
Source: PLoS Biol. 2025 Nov 4;23(11):e3003484. doi: 10.1371/journal.pbio.3003484 (PMC12604801; doi:10.1371/journal.pbio.3003484)

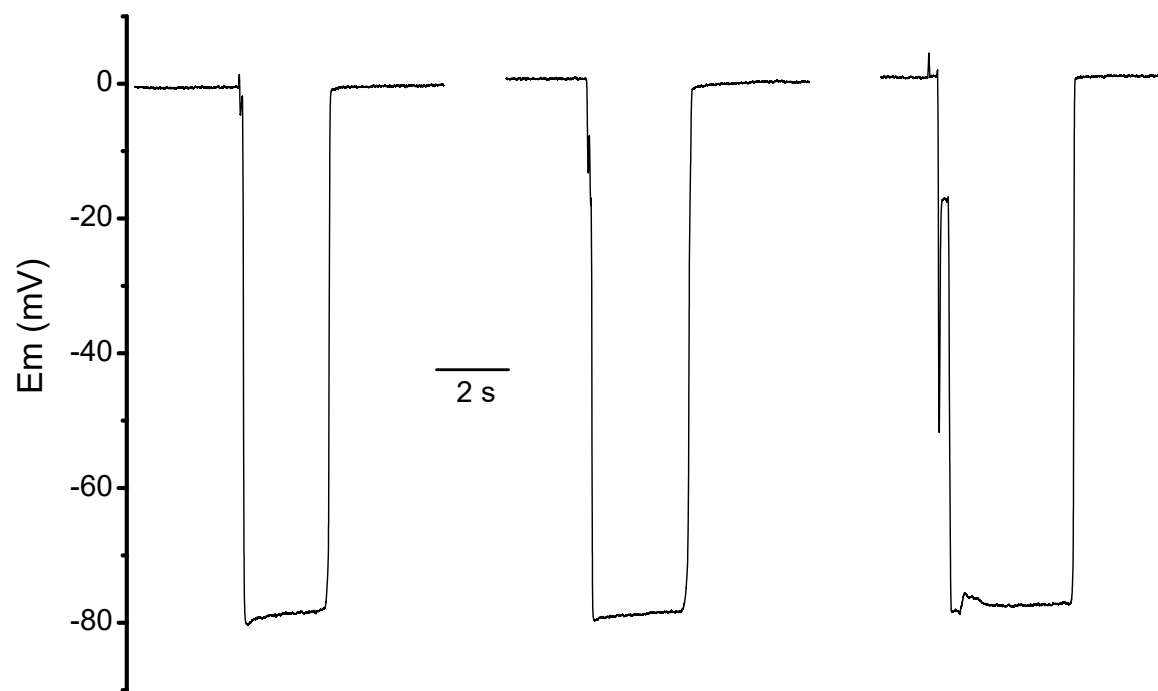

Figure S1

Supplement: S1 Fig — Resting membrane potential was measured in situ in skeletal muscle fibers in euthanized WT zebrafish using intracellular microelectrodes and a micro-electrode amplifier (VF 1800, BioLogic, France) in the presence of an extracellular Tyrode solution containing 2 mM K+ and an intramicroelectrode medium containing 3 M KCl. The microelectrodes resistance was between 20 and 30 MΩ. Measurements were done on muscle fibers located in the deep part of the trunk accessible to microelectrodes from the interior of the fish after having removed the internal organs and a thin film lining the interior of the thorax. All the fibers are fast-twitch type in this trunk region. Three different fibers were impaled with the same microelectrode that was slightly displaced laterally between each impalement. The mean resting membrane potential was found to be −78 ± 0.8 mV in 124 fibers from 4 fish. The data underlying S1 Fig can be found in S1 Data. (PDF) [file pbio.3003484.s001.pdf]

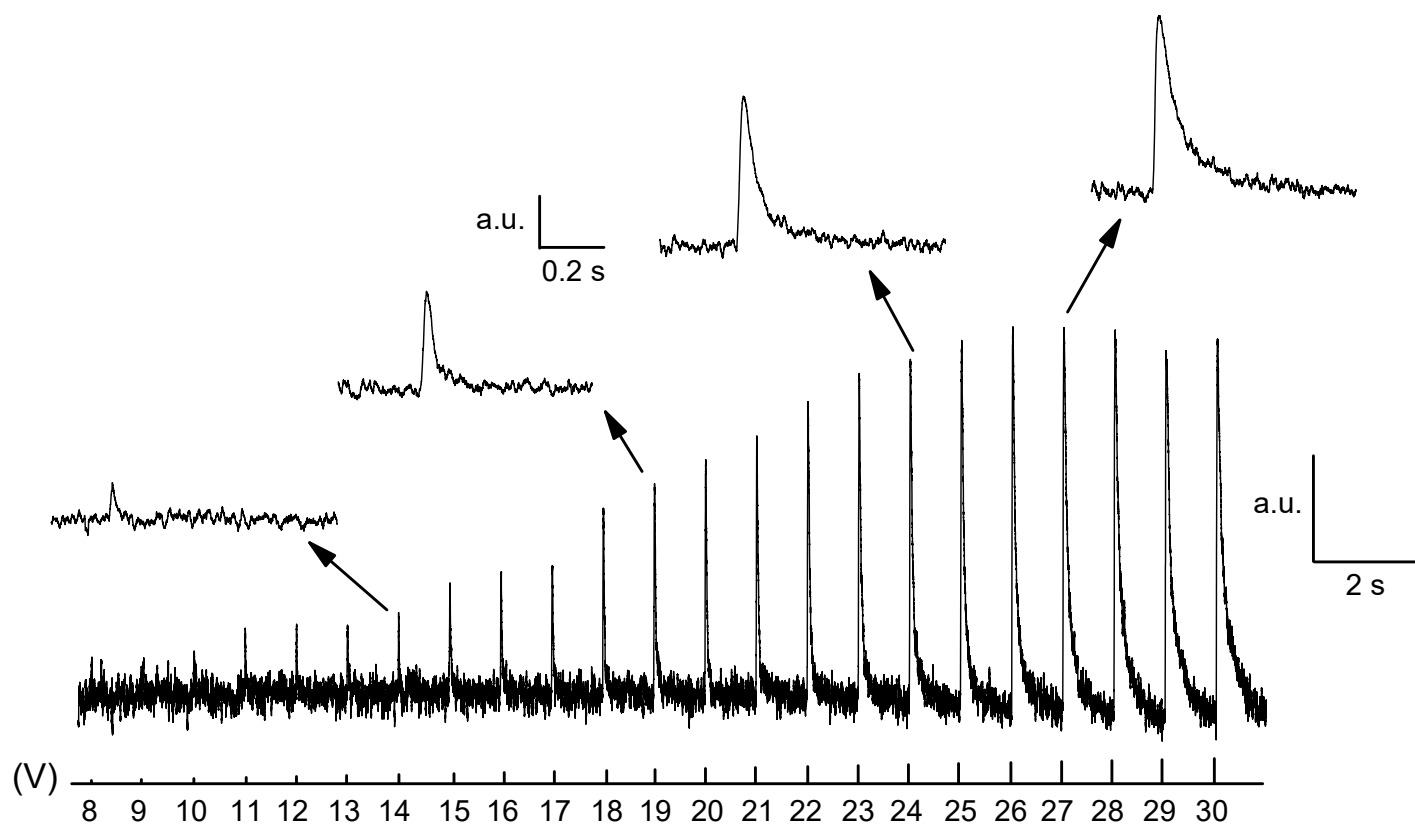

Figure S2

Supplement: S2 Fig — Fibers were loaded with the acetoxymethyl ester form of the Ca2+ fluorophore indo-1 (2 μM; Invitrogen) during 30 min and then subsequently washed (30 min) in Tyrode solution to allow de-esterification of the dye. Indo-1 fluorescence was measured on an inverted Nikon Diaphot epifluorescence microscope equipped with a commercial optical system, allowing the detection of fluorescence at 485 nm by a photomultiplier (IonOptix, Milton, MA, USA) upon 360 nm excitation. Fluorescence signals were acquired at a sampling frequency of 10 kHz. Fibers were electrically stimulated through parallel platinum field electrodes at 1 Hz with pulses of 0.5 ms duration. The voltage was gradually increased from 8 to 30 V, as indicated by the numbers below the lower trace. Arrows point to Ca2+ signals presented on an expanded scale. The stimulation frequency was 1 Hz, and the pulses duration was 0.5 ms. Graded responses to increasing voltages were observed in all the 8 tested fibers from 2 WT fish. (PDF) [file pbio.3003484.s002.pdf]

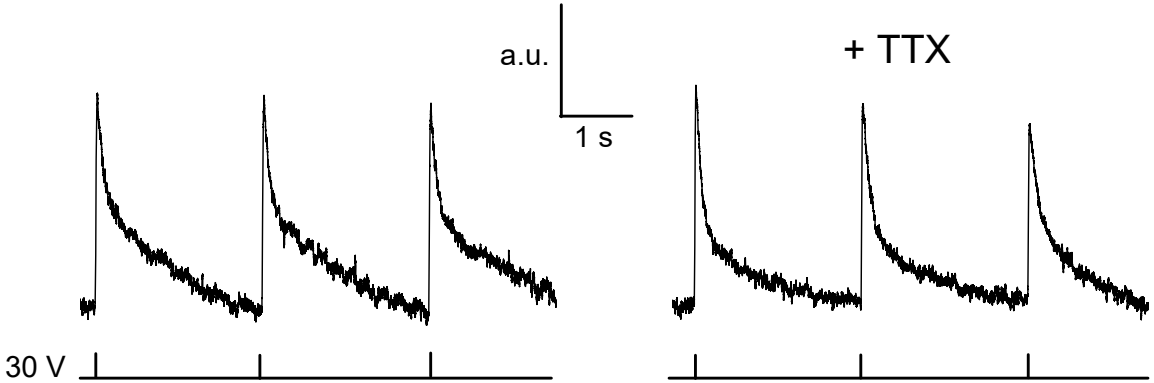

Figure S3

Supplement: S3 Fig — Changes in indo-1 fluorescence were measured using the same procedures as the ones indicated in the legend of S2 Fig in the absence (left) and after addition (right) of 2 µM TTX in the same fiber. Fibers were electrically stimulated at 0.4 Hz with pulses of 30 V amplitude and 0.5 ms duration. Absence of effect of TTX was observed in all the 10 fibers from 3 WT fish tested with this protocol. (PDF) [file pbio.3003484.s003.pdf]

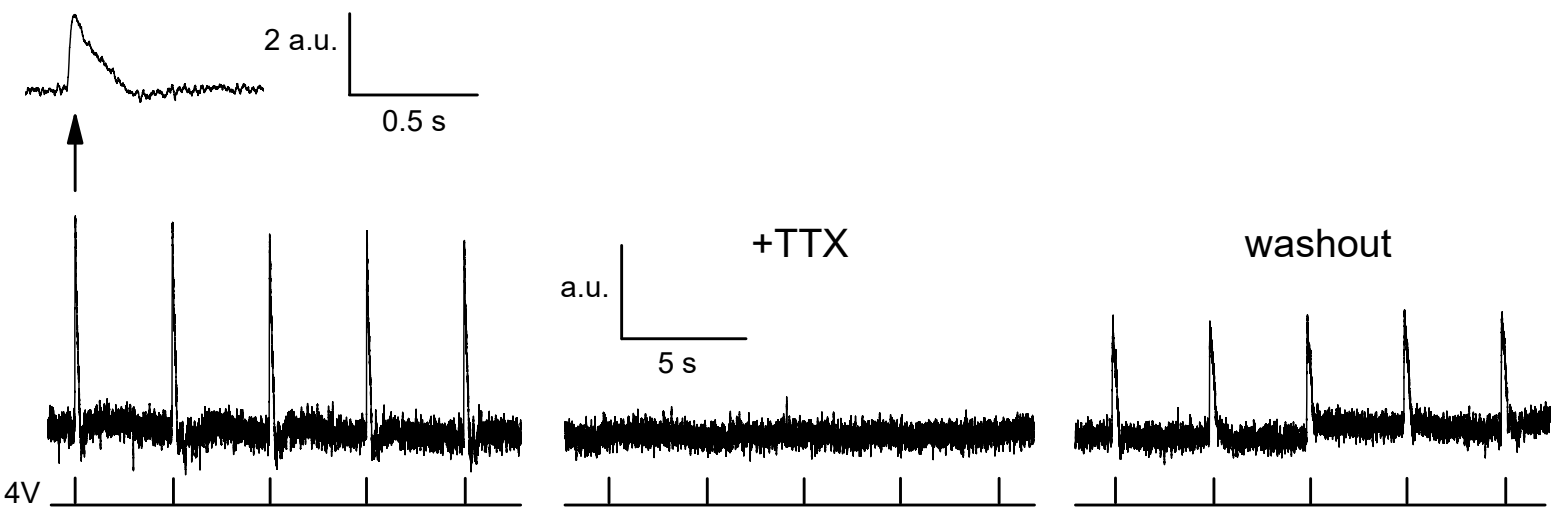

Figure S4

Supplement: S4 Fig — Changes in indo-1 fluorescence were measured using the same procedures as the ones indicated in the legend of S2 Fig in the absence (left), in the presence (middle), and after washout (right) of 2 µM TTX in the same fiber. The stimulation frequency was 0.25 Hz, and the pulses duration was 0.5 ms. Note that all-or-none Ca2+ signals were evoked in response to pulses of lower voltage (4 V) as compared to zebrafish. The arrow points to Ca2+ signal presented on an expanded scale. An inhibitory effect of TTX was observed in all the 10 tested fibers from 2 mice. (PDF) [file pbio.3003484.s004.pdf]

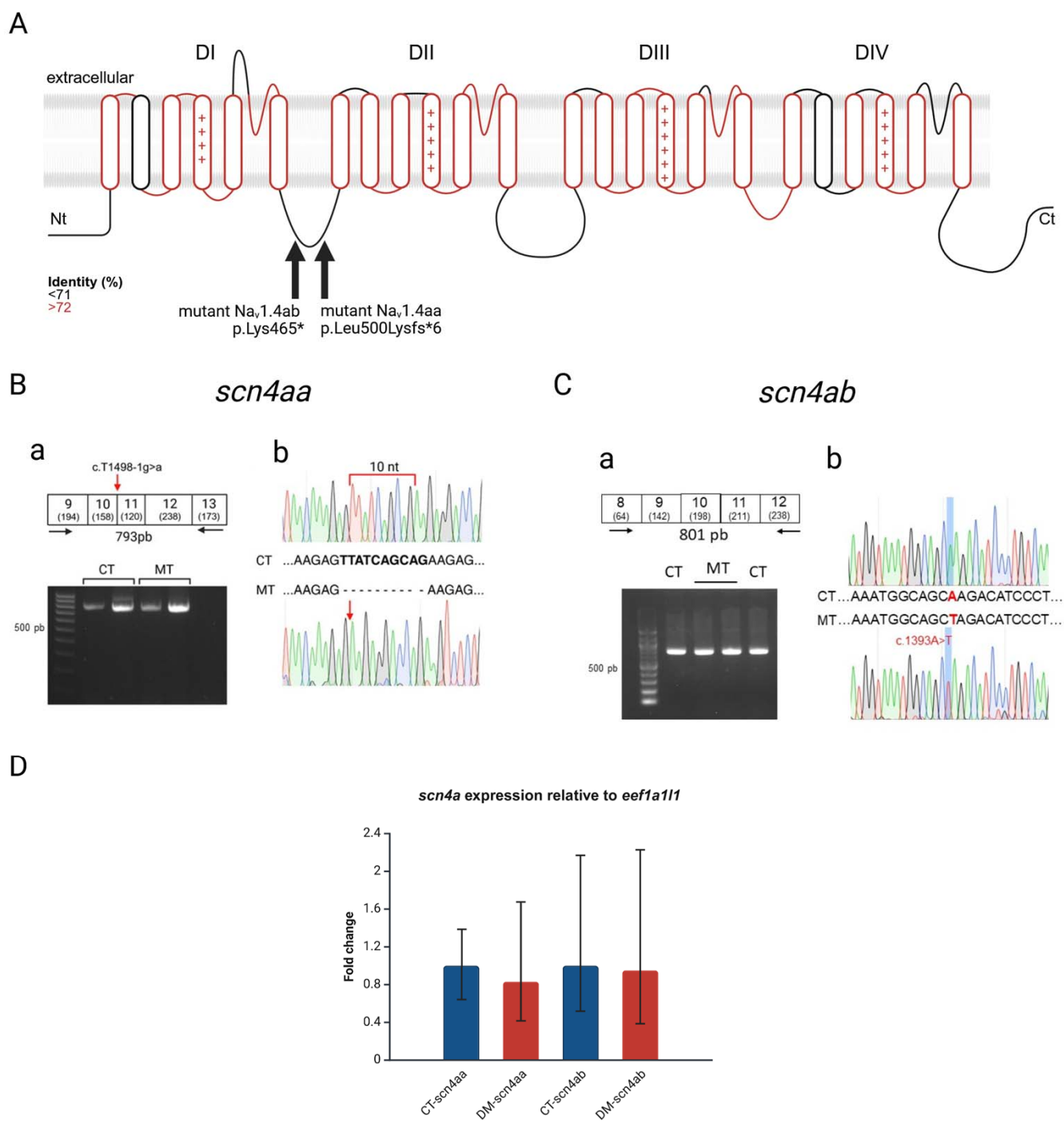

Figure S7

Supplement: S7 Fig — (A) Schematic representation for NaV1.4a inserted into the membrane with colors indicating the degree of identity between mammalian and zebrafish NaV1.4a proteins. The position of the scn4aa (NaV1.4aa) and scn4ab (NaV1.4ab) mutations is indicated. (B) Effect of the c.T1498-1g>a splicing mutation on scn4aa mRNA. (a) This point mutation is located in the acceptor splice site of intron 10 where it substitutes the invariable G nucleotide of the highly conserved AG sequence by an A. The PCR primers used to investigate its effect on scn4aa mRNA splicing are located in exons 9 and 13 (arrows). A single amplicon at the expected size (793 pb) was obtained when amplifying scn4aa cDNAs from adult skeletal muscles of zebrafish homozygous for the mutation (MT), similar to control animals from the same lays (CT). Left well: 100 pb ladder. (b) Sanger sequencing electropherograms of the PCR amplicons obtained from CT and MT muscle samples showed that the mutant scn4aa cDNA is deleted for 10 nucleotides (in bold) in the 5′ region of exon 11 (red horizontal bar and vertical arrow in the CT and MT electropherograms, respectively). A cryptic acceptor splice site (AG motif in the 3′ region of the deleted sequence) is used for intron 10 splicing in mutant scn4aa mRNA. (C) Effect of the c.1393A > T nonsense mutation on scn4ab mRNA. (a) A single amplicon at the expected size (800 pb) was obtained when amplifying scn4ab cDNAs from adult skeletal muscles of zebrafish homozygous for the mutation (MT) with PCR primers located in exons 8 and 12 (arrows). Left well: 100 pb ladder. (b) Sanger sequencing electropherograms of the PCR amplicons obtained from homozygous CT and MT muscle samples showed that all the mutant scn4ab cDNAs contain the nonsense mutation. (D) Quantitative real-time PCR analyses of scn4aa and scn4ab gene expression in trunk muscles of control and mutant adult fish did not argue for a decreased amount of scn4a mRNAs in double mutant (DM) samples compared to control (CT) sample [file pbio.3003484.s007.pdf]

**A**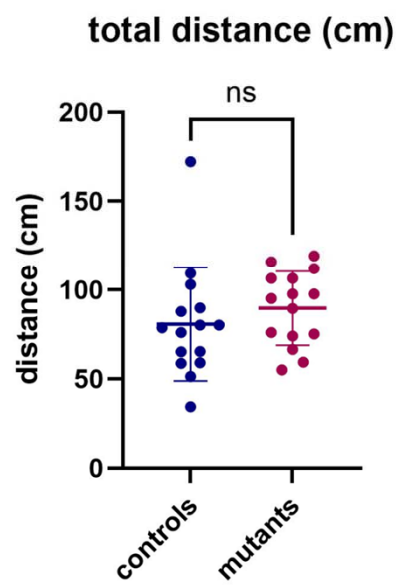**B**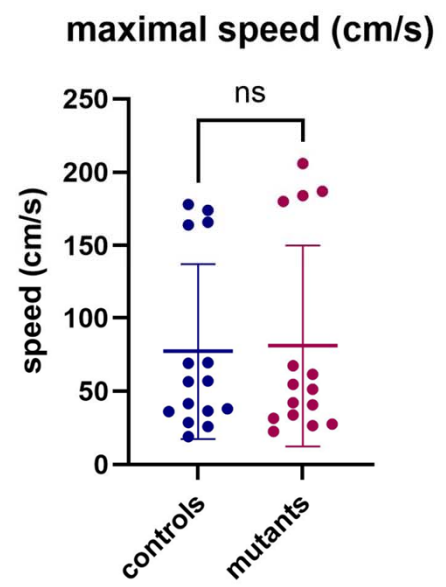

Figure S8

Supplement: S8 Fig — (A) Total distance covered in 10 s. (B) Maximal speed. Graphs represent mean ± SD with individual values (unpaired Mann–Whitney test) (4 to 12-month-old, minimum of 10 fish per genotype). The data underlying S8A and S8B Fig can be found in S1 Data. (PDF) [file pbio.3003484.s008.pdf]

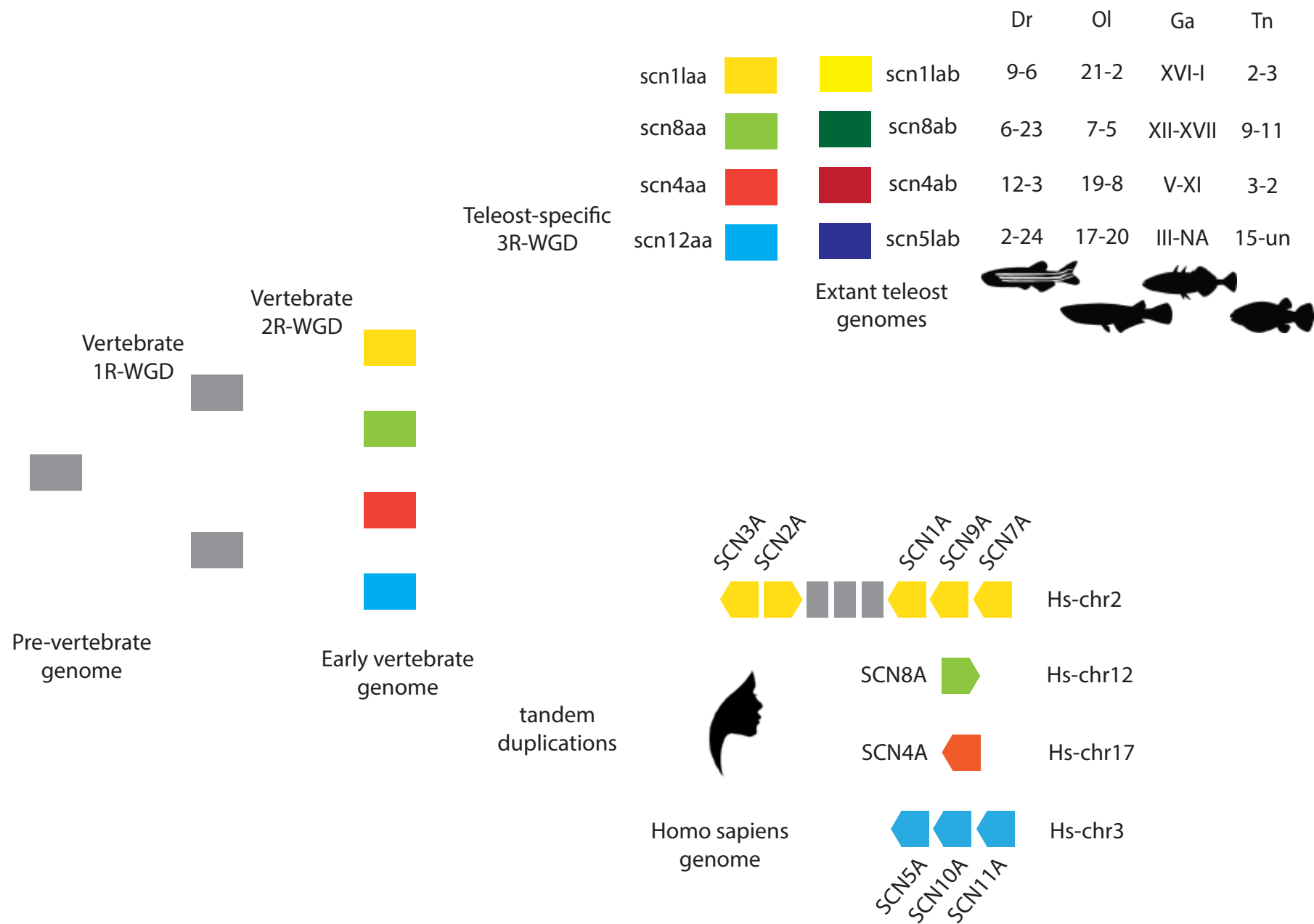

Figure S9

Supplement: S9 Fig — All the SCNA genes observed in vertebrates derive from a single SCNA gene of their latest common ancestor (gray). This gene went first through 2 rounds of whole-genome duplications (WGDs) to lead to 4 SCNA genes in the early vertebrate genome. The SCNA gene repertoire amplified furthermore, but in different modes of duplications according to genome of fish and mammalian lineages. A third WGD specific to the teleost fish lineage leads to 8 scna genes in those fish, while in human, tandem single gene duplications led to two clusters: one composed of SCN5A, SCN10A, SCN11A, and a second cluster containing SCN3A, SCN2A, SCN1A, SCN9A, and SCN7A. SCN4A and SCN8A do not show any further tandem duplications. Dr: Danio rerio (zebrafish; Cypriniformes, Ostariophysii); and three Percomorpha (Neoteleostei) Ol: Oryzias latipes (medaka); Ga: Gasterosteus aculeatus (stickleback); and Tn: Tetraodon nigroviridis (green spotted puffer). Chromosome numbers (or scaffold for Ga) are provided, unless not available (NA) or unassigned (un). In the human genome, the two clusters found on chromosome 2 are separated by three genes (gray boxes); Arrows represent the gene orientation (as depicted in Genomicus [38]). (Free silhouette images of organisms are from PhyloPic, version 2.0 (https://www.phylopic.org/nodes)). (PDF) [file pbio.3003484.s009.pdf]
