# Supplementary material for: Zebrafish fast muscle contractions avoid the mammalian requirement for voltage-gated Na+ channels
Source: PLoS Biol. 2025 Nov 4;23(11):e3003484. doi: 10.1371/journal.pbio.3003484 (PMC12604801; doi:10.1371/journal.pbio.3003484)
Supplement: S5 Fig — PCR amplification was done using cDNA samples obtained by reverse-transcription of total RNA samples extracted from trunk muscles of control (C) and digenic scn4aa−/−;scn4ab−/− mutant (DM) adult fish (3 fish by genotype) and control (L+) and digenic mutant (L−) 6-days-post fertilization(dpf) larvae (pool of 30 larvae). Genomic DNA (D, 25 ng) was used to demonstrate the absence of genomic DNA contamination in cDNA samples (no PCR products or PCR products of higher size), and water (0) to demonstrate the absence of any PCR contamination. The eef1a1l1 gene was used as control of reverse-transcription efficiency. Primers used are listed in S2 Table. A touch-down protocol was used for PCR amplification. A single amplicon at the expected size was obtained for all genes in the larval samples as expected, since whole larvae were used for RNA extraction. In trunk muscle extracts of control and double mutant samples, PCR amplicons were only obtained for scn4aa and scn4ab, confirming that scn4a are the sole scna isoforms expressed in control muscles of zebrafish and suggesting the lack of upregulation of another scna gene expression in muscles of double mutant fish. Created in BioRender. NICOLE, S. (2025) https://BioRender.com/ddf4c4j. (PDF) [file pbio.3003484.s005.pdf]

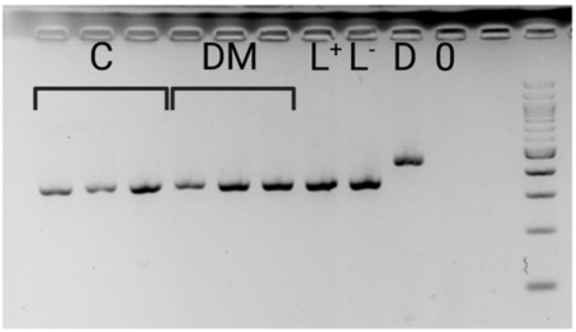


***eef1a1l1* (357 bp)**

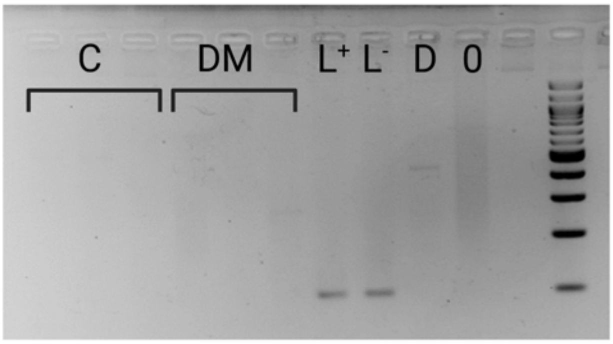


***scn5laa* (100 bp)**

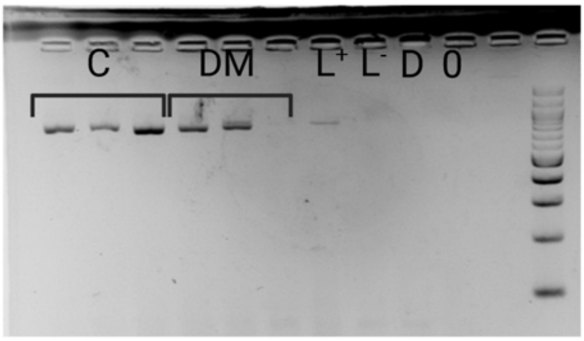


***scn4aa* (793 bp)**

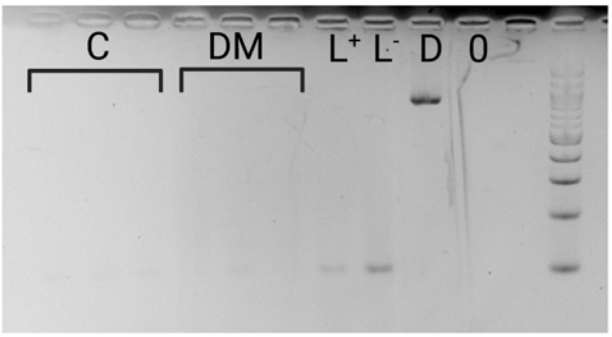


***scn5lab* (105 bp)**

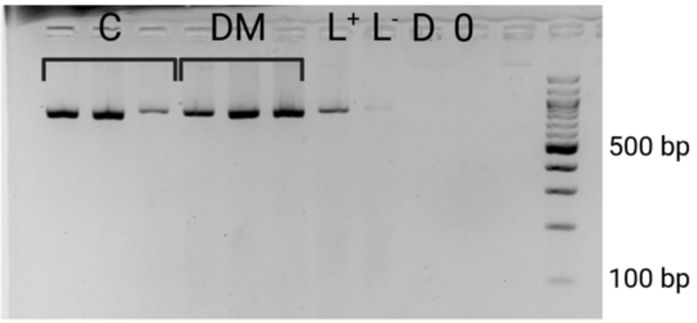


***scn4ab* (702 bp)**

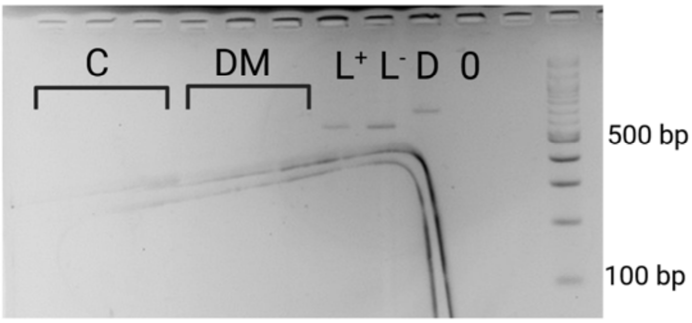


***scn8aa* (546 bp)**

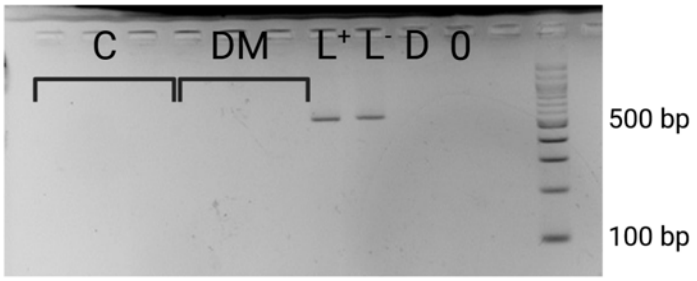


***scn1laa* (565 bp)**

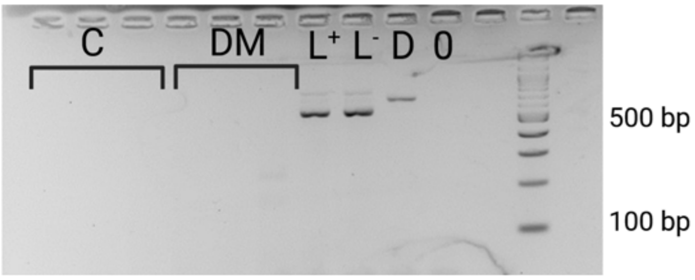


***scn8ab* (560 bp)**

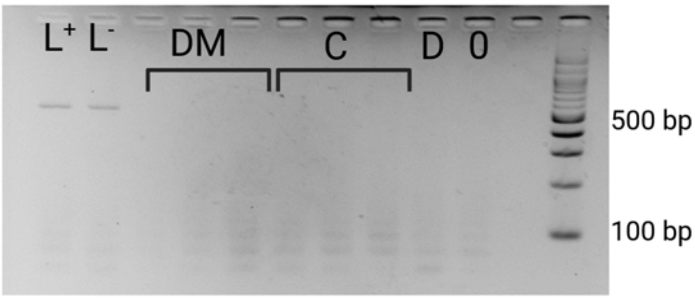


***scn1lab* (527 bp)**

C: controls (*scn4aa*$^{+/+}$; *scn4ab*$^{+/+}$)
DM: double mutants (*scn4aa*$^{-/-}$; *scn4ab*$^{-/-}$)
$L^+$ : pool of 30 6-dpf-old larvae, controls
$L^-$: pool of 30 6-dpf-old larvae, double mutants
D: genomic DNA (wild type fish, AB line)
O : negative control (water)
100 bp ladder

Figure S5
