# Supplementary material for: Zebrafish fast muscle contractions avoid the mammalian requirement for voltage-gated Na+ channels
Source: PLoS Biol. 2025 Nov 4;23(11):e3003484. doi: 10.1371/journal.pbio.3003484 (PMC12604801; doi:10.1371/journal.pbio.3003484)
Supplement: S6 Fig — Amino acids conserved between mouse and zebrafish proteins are in red. The 6 transmembrane segments (S) of the 4 domains (D) are underlined in yellow, the pore-forming loops are underlined in gray, and the inactivation gate is underlined in green. (PDF) [file pbio.3003484.s006.pdf]

```
hsNav1.4a     1     MARPSLCTLVPLGPECLRPFTRESLAAIEQRAVEEEARLQRNKQ------ME-IEEPERKPRSDLEAGKNLPMIYGDPPP   73
mmNav1.4a     1     MASSSLPTLVPPGPHCLRPFTPESLAAIEQRAMEEEARLQRNKQ------ME-IEEPERKPRSDLEAGKNLPLIYGDPPP   73
drNav1.4aa    1     MARL----LPPTGTSVFRRFTPESLVEIERLIQEKSTR----EE------LEGAEEEPQAPSSDLEAGKCLPMIYGDPPG   66
drNav1.4ab    1     MARL----LPPTGTDVFRPLTLESLAEIDRRMAEEAAE----QErmkeqnVKVAEEDLPKPTSDLEAGKVLPFIYGDPPP   72

                                                                                 DIS1
hsNav1.4a     74    EVIGIPLEDLDPYYSNKKTFIVLNKGKAIFRFSATPALYLLSPFSVVRRGAIKVLIHALFSMFIMITILTNCVFMTMSDP   153
mmNav1.4a     74    EVIGVPLEDLDPYYSDKKTFIVLNKGKAIFRFSATPALYMLSPFSIVRRVAIKVLIHALFSMFIMITILTNCVFMTMSNP   153
drNav1.4aa    67    DLLNTPLEDIDPFYKTQKTFIVISKGNTIFRFSSEPAMFCISPFSIVRRGAIKILIHSLFSMFIMITILSNCVFMTMSNP   146
drNav1.4ab    73    NLLNVPIEELDPYYKAQKTFIVIDKKNTIYRFNTEPACYCLSPFNPVRRAAIRILIHSLFSLVIMLTILTNCVFMAMSDP   152

                       DIS2                            DIS3                    DIS4
hsNav1.4a     154   PPWSKNVEYTFTGIYTFESLIKILARGFCVDDFTFLRDPWNWLDFSVIMMAYLTEFVDLGNISALRTFRVLRALKTITVI   233
mmNav1.4a     154   PSWSKDVEYTFTGIYTFESLIKMLARGFCIDDFTFLRDPWNWLDFSVITMAYVTEFVDLGNISALRTFRVLRALKTITVI   233
drNav1.4aa    147   PAWSKTVEYVFTGIYTFEATVKVLSRGFCIGPFTFLRDPWNWLDFMVISMAYVTEFVDLGNVSALRTFRVLRALKTITVI   226
drNav1.4ab    153   PGWSKILEYVFTGIYTFEAMVKVLSRGFCIGDFTFLRDPWNWLDFMVISMAYLTEFVDLGNISALRTFRVLRALKTITVI   232

                                   DIS5
hsNav1.4a     234   PGLKTIVGALIQSVKKLSDVMILTVFCLSVFALVGLQLFMGNLRQKCVRWPPPFNDT-----NTTWYSNDTWYGNDTWYG   308
mmNav1.4a     234   PGLKTIVGALIQSVKKLSDVMILTVFCLSVFALVGLQLFMGNLRQKCVRWPPPMNDT-----NTTWYGNDTWYGNDTWYG   308
drNav1.4aa    227   PGLKTIVGALIQSVKKMIDVMILTIFALAVFALIGLQLFMGNLRQKCIRWPI----L-----NSTIF--DVYNSNMV---   292
drNav1.4ab    233   PGLKTIVGALIQSVKKLADVMILTVFCLSVFALIGLQLFMGNLRQKCVLWPP----VgwysdNLTVL--SNYTDING---   303

hsNav1.4a     309   NEMWYGNDswyandTWNSHASWATNDTFDWDAYISDEGNFYFLEGSNDALLCGNSSDAGHCPEGYECIKTGRNPNYGYTS   388
mmNav1.4a     309   NDTWYGND------TWNSQESWVSNSTFDWEAYINDEGNFYFLEGSNDALLCGNSSDAGHCPEGYECMKAGRNPNYGYTS   382
drNav1.4aa    293   NDT-----------TLNVTD------TFDFKAYINNEENQYFLEGSLDALLCGNSSDAGRCPEGYTCMKAGRNPNYGYTS   355
drNav1.4ab    304   NGT-----------A---NS------TFDYQKYINSEENYYYVPGQMDPLVCGNSSDAGLCPEGYICLKAGRNPNYGYTS   363

                      Pore-forming loop, DI          DIS6
hsNav1.4a     389   YDTFSWAFLALFRLMTQDYWENLFQLTLRAAGKTYMIFFVVIIFLGSFYLINLILAVVAMAYAEQNEATLAEDKEKEEEF   468
mmNav1.4a     383   YDTFSWAFLALFRLMTQDYWENLFQLTLRAAGKTYMIFFVVIIFLGSFYLINLILAVVAMAYAEQNEATLAEDQEKEEEF   462
drNav1.4aa    356   YDNFGWAFLALFRLMTQDFWENLFQLTLRAAGKTYMIFFVVVIFLGSFYLINLILAVVAMAYDEQNEATLAEARDKEEEF   435
drNav1.4ab    364   YDNFGWAFLALFRLMTQDFWENLFQLTLRAAGKTYMIFFVVIIFLGSFYLINLILAVVAMAYAEQNEATAAEAKEKEEEY   443

hsNav1.4a     469   QQMLEKFKKHQ-------EELEKAKAAQALEG-GE-ADG------DPAHGKDCNGSL-------------DTSqGEKGAP   520
```

Figure S6

```
mmNav1.4a    463   QQMLEKFKKHQ-------EELEKAKAAQALEG-GEEADG------DPTHSKDCNGSL-------------DTS-GEKGPP   514
drNav1.4aa   436   QRLLEQLKNQE-------TGSKASLASQKTQSRGSNRTGslhdlaDEDVIKDCNGRIVPRLivnrvssnkELS-AEEDQK   507
drNav1.4ab   444   AKIMEQLKKQAeqkngmvNGSKTSLSSKK---KGDN----------DQMQSDYDGIALKPL-----------------SK   493

                                                                                      DIIS1
hsNav1.4a    521   -----------------RQSSSGDSGISDAMEELEEAHQKCPPWWYKCAHKVLIWNCCAPWLKFKNIIHLIVMDPFVDLG   583
mmNav1.4a    515   -----------------RPSCSAESAISDAMEELEEAHQKCPPWWYKCAHKVLIWNCCAPWVKFKHIILLIVMDPFVDLG   577
drNav1.4aa   508   SLSSKHSMQYLDQPKLSKRTASALSVLTATMEGLEDAQRPCPPGWYKFADMFLKWDCCAPWILFKKWVHFVVMDPFVDLG   587
drNav1.4aa   494   SNGSKGNINYLEVPDSQIRKPSVVSAVESALDAQEDIERPCPPGWYKFADIFLKWDCCIPWVKFKRIVYLFVMDPFVDLG   573

                                           DIIS2                              DIIS3
hsNav1.4a    584   ITICIVLNTLFMAMEHYPMTEHFDNVLTVGNLVFTGIFTAEMVLKLIAMDPYEYFQQGWNIFDSIIVTLSLVELGLANVQ   663
mmNav1.4a    578   ITICIVLNTLFMAMEHYPMTEHFDNVLSVGNLVFTGIFTAEMVLKLIAMDPYEYFQQGWNIFDSFIVTLSLVELGLANVQ   657
drNav1.4aa   588   ITICIVLNTLFMAMEHYPMSPHFEHVLSVGNLVFTGIFTAEMVFKLIAMDPYYYFQVGWNIFDSIIVTLSLVELGLANVQ   667
drNav1.4aa   574   ITLCIVLNTVFMAMEHYPMSVHVEEVLAIGNLVFTGIFAAEMVLKLIALDPYYYFQVGWNIFDSIIVTMSLVELMLADVE   653

                    DIIS4                              DIIS5
hsNav1.4a    664   GLSVLRSFRLLRVFKLAKSWPTLNMLIKIIGNSVGALGNLTLVLAIIVFIFAVVGMQLFGKSYKECVCKIALDCNLPRWH   743
mmNav1.4a    658   GLSVLRSFRLLRVFKLAKSWPTLNMLIKIIGNSVGALGNLTLVLAIIVFIFAVVGMQLFGKSYKECVCKIASDCSLPRWH   737
drNav1.4aa   668   GLSVLRSFRLLRVFKLAKSWPTLNMLIKIIGNSVGALGNLTLVLAIIVFIFAVVGMQLFGKSYKDCVCKISEDCELPRWH   747
drNav1.4aa   654   GLSVLRSFRLMRVFKLAKSWPTLNMLIKIIGNSVGALGNLTLVLAIIVFIFAVVGMQLFGKSYTDSVCKISSDCELPRWH   733

                      Pore-forming loop, DII           DIIS6
hsNav1.4a    744   MHDFFHSFLIVFRILCGEWIETMWDCMEVAGQAMCLTVFLMVMVIGNLVVLNLFLALLLSSFSADSLAASDEDGEMNNLQ   823
mmNav1.4a    738   MHDFFHSFLIVFRILCGEWIETMWDCMEVAGQAMCLTVFLMVMVIGNLVVLNLFLALLLSSFSADSLAASDEDGEMNNLQ   817
drNav1.4aa   748   MNDFFHSFLIVFRILCGEWIETMWDCMEVAGASMCLIVFMMVMVIGNLVVLNLFLALLLSSFSGDNLSGGDDDGEMNNLQ   827
drNav1.4ab   734   MADFFHAFLIIFRVLCGEWIETMWDCMEVAGQGMCIIVFMMVMVIGNLVVLNLFLALLLSSFSGDNLSASDDDGE-NNLQ   812

hsNav1.4a    824   IAIGRIKLGIGFAKAFLLGLLHGKILSPKDIMLSLGEADGageAGEAGETAP--EDEKKEPPEEDLK---KDNHILNHMG   898
mmNav1.4a    818   IAIGRIKWGIAFAKTFLLGLLHGKILSLKDIMLSLGEPGG---AGENGESPP--EDEKKEPPPEDGNKELKDNHILNHVG   892
drNav1.4aa   828   IAIGRITRGIDWVKALVASMVQ-RILGKK----------------------P--DNTKEEG---EGDIEL---YALNH--   874
drNav1.4ab   813   IAISRITRGIDWIKAFVNKHVR-QCLNLK----------------------PkeEGAKVNG---EGDAKM---NAI----   859

hsNav1.4a    899   LADGPPSSLELDHLNFINNPYLTIQVPIASEESDLEMPTEEETDTFSEPEDSKKPPQ-PLYDGN-SSVCSTADYKPPEED   976
mmNav1.4a    893   LTDGPRSSIEMDHLNFINNPYLTIHVPIASEESDLEMPTEEETDTFSEPEDIKKPLQ-PLYDGN-SSVCSTADYKPPEED   970
```

Figure S6

```
drNav1.4aa   875   LDEGKMA----DGLTNCLSPTLT--VPIARCESDVE---EDEDSESSDEEDAKATLN----DGD-SSVCSTVDYQPPEPE   940
drNav1.4ab   860   --------------MNSSSSMVK--VPIANGESD-D---DDGNGSSEDEDDEGRDINmKKKNGDeSSTCSTVD-KPPEVE   918

                                                                                  DIIIS1
hsNav1.4a    977   PE--EQAEENPEGEQPEECFTEACVQRWPCLYVDISQGRGKKWWTLRRACFKIVEHNWFETFIVFMILLSSGALAFEDIY   1054
mmNav1.4a    971   PE--EQAEENPEGELPEECFTEACVKRCPCLYVDISQGRGKMWWTLRRACFKIVEHNWFETFIVFMILLSSGALAFEDIY   1048
drNav1.4aa   941   PEpEEVEEEEPEPEEPEACFTEGCIRRCACLSVDITEGWGKKWWNLRRTCFTIVEHDYFETFIIFMILLSSGALAFEDIN   1020
drNav1.4ab   919   ---DLVEEEEEDLTSPEDCYTENCIRRCPCLDLDVSQGKGKAWWNFRKTCFAIVEHSYFETFIIFMILLSSGALAFEDIY   995

                             DIIIS2                          DIIIS3                    DIIIS4
hsNav1.4a    1055  IEQRRVIRTILEYADKVFTYIFIMEMLLKWVAYGFKVYFTNAWCWLDFLIVDVSIISLVANWLGYSELGPIKSLRTLRAL   1134
mmNav1.4a    1049  IEQRRVIQTILEYADKVFTYIFILEMLLKWVAYGFKVYFTNAWCWLDFLIVDVSIISLVANWLGYSELGPIKSLRTLRAL   1128
drNav1.4aa   1021  IERRRVIKTILEYADKVFTYIFIVEMLLKWVAYGFKTYFTNAWCWLDFLIVDVSLVSLTANLMGYSELGAIKSLRTLRAL   1100
drNav1.4ab   996   IEQRRMIKIILEYADQVFTYVFVVEMLLKWVAYGFKVYFTNAWCWLDFLIVDVSLISLTANILGYSELGAIKSLRTLRAL   1075

                                          DIIIS5
hsNav1.4a    1135  RPLRALSRFEGMRVVV-NALLGAIPSIMNVLLVCLIFWLIFSIMGVNLFAGKFYYCINTTTSERFDISEVNNKSECESLM   1213
mmNav1.4a    1129  RPLRALSRFEGMRVVV-NALLGAIPSIMNVLLVCLIFWLIFSIMGVNLFAGKFYYCINTTTSERFDISVVNNKSECESLM   1207
drNav1.4aa   1101  RPLRALSRFEGMRVVV-NALVGAIPSIFNVLLVCLIFWLIFSIMGVNLFAGKFYHCINTTTEERIPMDVVNNKSDCMALM   1179
drNav1.4ab   1076  RPLRALSRFEGMRVVVvNALVGAIPSIFNVLLVCLIFWLIFSIMGVNLFAGKFYYCFNETSEEVFDHNVVNNKTDCYELM   1155

                                   pore forming loop, DIII                   DIIIS6
hsNav1.4a    1214  -HTGQVRWLNVKVNYDNVGLGYLSLLQVATFKGWMDIMYAAVDSREKEEQPQYEVNLYMYLYFVIFIIFGSFFTLNLFIG   1292
mmNav1.4a    1208  -YTGQVRWMNVKVNYDNVGLGYLSLLQVATFKGWMDIMYAAVDSREKEEQPDYEVNLYMYLYFVIFIIFGSFFTLNLFIG   1286
drNav1.4aa   1180  -YTNEVRWVNVKVNYDNVGLGYLSLLQIATFKGWMDIMYAAVDSREVDEQPSYEINLYMYLYFVIFIIFGSFFTLNLFIG   1258
drNav1.4ab   1156  eFHPEVRWMNGKINFDNVGMGYLALLQVATFKGWMDIMYSAVDSRAIESQPVYEANLYMYIYFVIFIIFGSFFTLNLFIG   1235

                                  Inactivation gate/ Pan Nav epitope                 DIVS1
hsNav1.4a    1293  VIIDNFNQQKKKLGGKDIFMTEEQKKYYNAMKKLGSKKPQKPIPRPQNKIQGMVYDLVTKQAFDITIMILICLNMVTMMV   1372
mmNav1.4a    1287  VIIDNFNQQKKKFGGKDIFMTEEQKKYYNAMKKLGSKKPQKPIPRPQNKIQGMVYDFVTKQVFDISIMILICLNMVTMMV   1366
drNav1.4aa   1259  VIIDNFNQQKSKFGGKDIFMTEEQKKYYNAMKKLGAKKRPKPIPRPSNIIQGLVFDFISKQFFDIFIMVLICLNMVTMMI   1338
drNav1.4ab   1236  VIIDNFNQQKAKLGGTDIFMTEEQKKYYNAMKKLGSKKPQKPIPRPTNCCQGLVFDFVTQQFFDIFIMVMICLNMVTMMV   1315

                             DIVS2                           DIVS3                          DIVS4
hsNav1.4a    1373  ETDNQSQLKVDILYNINMIFIIIFTGECVLKMLALRQYYFTVGWNIFDFVVVILSIVGLALSDLIQKYFVSPTLFRVIRL   1452
mmNav1.4a    1367  ETDDQSQLKVDILYNINMVFIIVFTGECVLKMFALRHYYFTIGWNIFDFVVVILSIVGLALSDLIQKYFVSPTLFRVIRL   1446
```

Figure S6

```
drNav1.4aa   1339   ETDDQSAEKEYVLYQINLVFIVVFTSECVLKLFALRQYFFTIGWNVFDFVVVILSIAGLMLSDIIEKYFVSPTLFRVIRL   1418
drNav1.4ab   1316   ETDDQSAEIEEILFYINFAFIILFTGECVLKITALRYHYFSIGWNIFDFVVVILSILGIGLADLIEKYFVSPTLFRVIRL   1395

                                               DIVS5                                   pore forming loop, DIV
hsNav1.4a    1453   ARIGRVLRLIRGAKGIRTLLFALMMSLPALFNIGLLLFLVMFIYSIFGMSNFAYVKKESGIDDMFNFETFGNSIICLFEI   1532
mmNav1.4a    1447   ARIGRVLRLIRGAKGIRTLLFALMMSLPALFNIGLLLFLVMFIYSIFGMSNFAYVKKESGIDDMFNFETFGNSIICLFEI   1526
drNav1.4aa   1419   ARIGRVLRLIRGAKGIRTLLFALMMSLPALFNIGLLLFLIMFIFSIFGMSNFAYVKKQAGIDDIFNFETFGGSIICLFEI   1498
drNav1.4ab   1396   ARIGRVLRLIRGAKGIRTLLFALMMSLPALFNIGLLLFLIMFIFSIFGMSNFAYVKKEVGIDDMMNFETFGNSIICMFMI   1475

                                                                  DIVS6
hsNav1.4a    1533   TTSAGWDGLLNPILNSGPPDCDPNLENPGTSVKGDCGNPSIGICFFCSYIIISFLIVVNMYIAIILENFNVATEESSEPL   1612
mmNav1.4a    1527   TTSAGWDGLLNPILNSGPPDCDPTLENPGTNIKGDCGNPSIGICFFCSYIIISFLIVVNMYIAIILENFNVATEESSEPL   1606
drNav1.4aa   1499   TTSAGWDGLLLPILNSGPPDCDPDFENPGTDVRGNCGNPGMGIMFFCSYIIMSFLVVVNMYIAIILENFNNAQEESGDPL   1578
drNav1.4ab   1476   TTSAGWDGLLAPILNS-PPDCDPDVDNPGSTTRGNCGNAAVGIVFFCSYIVMSFLVVVNMYIAIILENFNVATEESSDPL   1554

hsNav1.4a    1613   GEDDFEMFYETWEKFDPDATQFIAYSRLSDFVDTLQEPLRIAKPNKIKLITLDLPMVPGDKIHCLDILFALTKEVLGDSG   1692
mmNav1.4a    1607   CEDDFEMFYETWEKFDPDATQFIDYSRLSDFVDTLQEPLKIAKPNKIKLITLDLPMVPGDKIHCLDILFALTKEVLGDSG   1686
drNav1.4aa   1579   CEDDFDMFDETWEKFDVDATQFIEYDRLFDFVDALQEPLRIAKPNRLKLISMDIPIVNGDKIHSQDILLAVTREVLGDTI   1658
drNav1.4ab   1555   CEDDFEMFYETWEKFDPTASQFIDYNRLSEFCDTLKDPLRIPKPNTLKLITMDIPMVTGDKIHCLDLLLALTGEVLGGSD   1634

hsNav1.4a    1693   EMDALKQTMEEKFMAANPSKVSYEPITTTLKRKHEEVCAIKIQRAYRRHLLQRSMKQASYMYRHSHD---GSGDDAPEKE   1769
mmNav1.4a    1687   EMDALKQTMEEKFMAANPSKVSYEPITTTLKRKQEEVCAIKIQRAYRRHLLQRSVKQASYMYRHSQE---GNGDGAPEKE   1763
drNav1.4aa   1659   EMDAMKESIEAKFIMNNPTSASFEPIITTLRRKEEERAAIAVQRIYRRHLLKRAIRYACFMRRSKRKVRNPNDNEPPETE   1738
drNav1.4ab   1635   QMDGMKATMEEKFMANNPSKASYEPITSTLKRKQEEVAASTIQRAYRSHILKRCVKQASYMYRDKTGSKKPT-GEAPEKV   1713

hsNav1.4a    1770   GLLANTMSKMYGHENG--------------NS--SSPSPEEKGEAGDAGPTMG---LMPISPSDTAW---PPAPPP----   1823
mmNav1.4a    1764   GLLANTMNKMYGSEKE--------------DNGVQSQGEKEKDSTEDAGPTTE---VTAPSSSDTALTPPPPSPPPPSSP   1826
drNav1.4aa   1739   GLIARKMNTLYGSNPElaMALEletrpmrpNSQPPKPSQVTQTRASVTFPRPQgqlILPVELTSEVILRSAPITHSLNSS   1818
drNav1.4ab   1714   GMIAENMRSLYGD-----QAVE--------DDHPVGCSFSQHGKTQFGAKRP------PVKVQSDVVLHSAPFPVP-ESS   1773

hsNav1.4a    1824   --GQTVRPGVKESLV   1836
mmNav1.4a    1827   PQGQTVRPGVKESLV   1841
drNav1.4aa   1819   ENATTI----KESIV   1829
drNav1.4ab   1774   TAADNL----RESIV   1784
```

Figure S6
