# Supplementary material for: Zebrafish fast muscle contractions avoid the mammalian requirement for voltage-gated Na+ channels
Source: PLoS Biol. 2025 Nov 4;23(11):e3003484. doi: 10.1371/journal.pbio.3003484 (PMC12604801; doi:10.1371/journal.pbio.3003484)
Supplement: S1 Table — (DOCX) [file pbio.3003484.s012.docx]

**S1 Table: Percent Identity Matrix between human, mouse and zebrafish Na_V_1.4a proteins**

|  | **Human** | **Mouse** | **Na_V_1.4aa** | **Na_V_1.4ab** |
| --- | --- | --- | --- | --- |
| **Human** | **100** | 93.10 | 71.80 | 70.50 |
| **Mouse** | 93.10 | **100** | 72.37 | 70.95 |
| **Na_V_1.4aa** | 71.80 | 72.37 | **100** | 72.44 |
| **Na_V_1.4ab** | 70.50 | 70.95 | 72.44 | **100** |
