# Supplementary material for: Zebrafish fast muscle contractions avoid the mammalian requirement for voltage-gated Na+ channels
Source: PLoS Biol. 2025 Nov 4;23(11):e3003484. doi: 10.1371/journal.pbio.3003484 (PMC12604801; doi:10.1371/journal.pbio.3003484)
Supplement: S2 Table — (DOCX) [file pbio.3003484.s013.docx]

**S2 Table: Primers used for analyzing gene expression by PCR**

| **Gene** | **primer 1 (5'-3')** | **primer 2 (5'-3')** | **Amplicon size (bp)** |
| --- | --- | --- | --- |
| *scn4aa* | GGCTCGTGACAAAGAAGAGG | GTTAAGGGTGGGCCAAGATTT | 793 |
| *scn4ab* | GGCTACACCAGTTATGACAAC | TAATATGGGTCCAGCGCA | 800 |
| *scn1laa* | GATTGAAATGACCCGTGCGC | GCCTACGACCGAACTTCCAG | 565 |
| *scn1lab* | GACAGAGGATATGGACAATA | AATCTGGATCTTGCTTGTTC | 527 |
| *scn5laa* | CACAGAACGCTCTCAGAGGA | GAGAGGAGAGTGTGGCCAA | 100 |
| *scn5lab* | CAACTGGGACCCCATAGAGG | CGGAAGTTGAAGATGCTGAC | 105 |
| *scn8aa* | ATCACAGGAGACGGAAAACA | GGATTTTCTTTAGTTGGCTC | 546 |
| *scn8ab* | TCAGACGAGACAGAGAACAT | TTAACTGTGGTTCCTGGGTT | 560 |
| *eef1a1l1* | CTTCTCAGGCTGACTGTGC | CCGCTAGCATTACCCTCC | 357 |
