# Supplementary material for: Zebrafish fast muscle contractions avoid the mammalian requirement for voltage-gated Na+ channels
Source: PLoS Biol. 2025 Nov 4;23(11):e3003484. doi: 10.1371/journal.pbio.3003484 (PMC12604801; doi:10.1371/journal.pbio.3003484)
Supplement: S1 Raw Images — (PDF) [file pbio.3003484.s014.pdf]

BMP files, agarose gel images (EtB staining) saved in inverted colors. Pictures taken with Genesmart apparatus (real-time images from 8 bit, 768 x 582 pixel camera)

FigS5	C	C	C	DM	DM	DM	L+	L-	D	0	x	100 bp ladder
FigS7	x	C	C	DM	DM	x	x	x	x	x	x	100 bp ladder

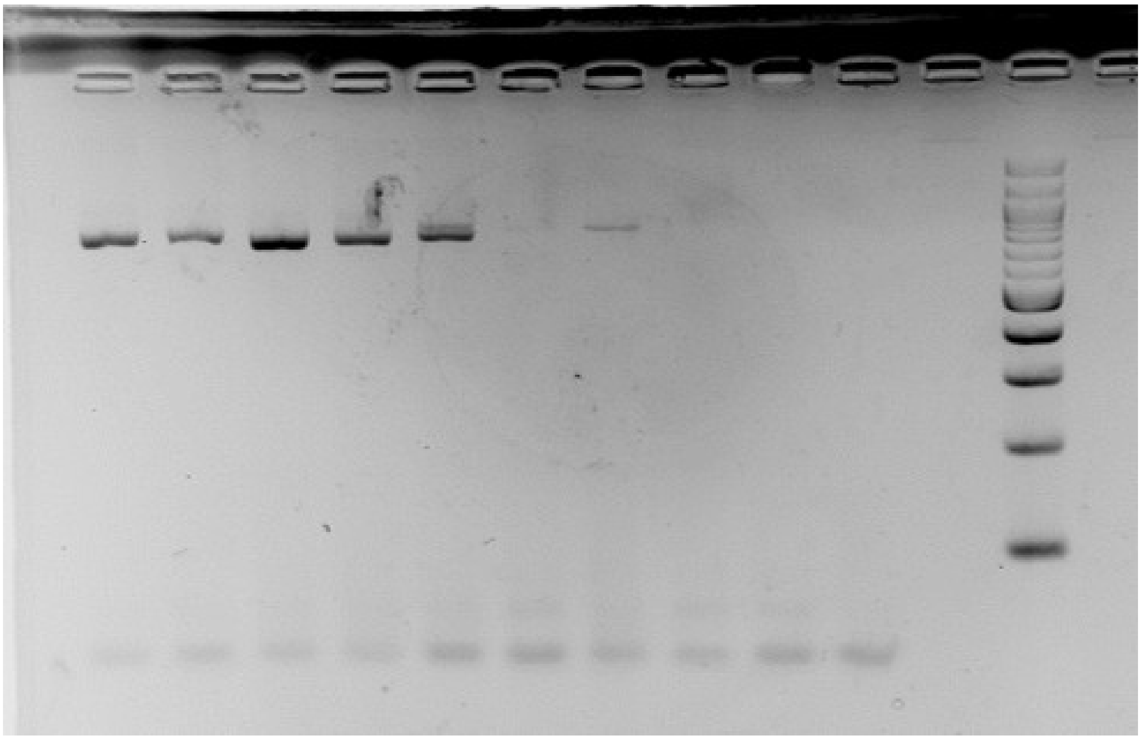

***scn4aa* (793 bp), Figures S5 and S7**

FigS5	C	C	C	DM	DM	DM	L+	L-	D	0	x	100 bp ladder
FigS7	C	C	x	x	DM	DM	x	x	x	x	x	100 bp ladder

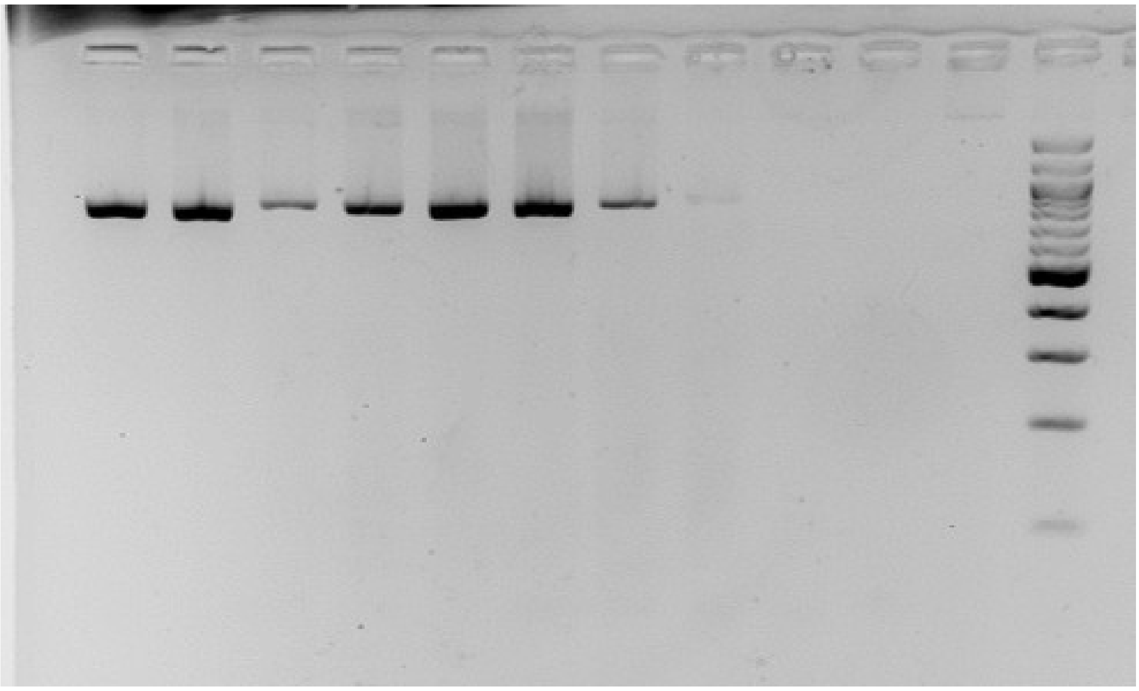

***scn4ab* (800 bp), Figures S5 and S7**

C: controls (*scn4aa*$^{+/+}$; *scn4ab*$^{+/+}$)
DM: double mutants (*scn4aa*$^{-/-}$; *scn4ab*$^{-/-}$)
L^{+} : pool of 30 6-dpf-old larvae, controls
L^{-}: pool of 30 6-dpf-old larvae, double mutants

D: genomic DNA (wild type fish, AB line)
O : negative control (water)
100 bp ladder
x: empty or not used in the final image

BMP files, agarose gel images (EtB staining) saved in inverted colors. Pictures taken with Genesmart apparatus (real-time images from 8 bit, 768 x 582 pixel camera)

C C C DM DM DM L+ L- D 0 x 100 bp ladder

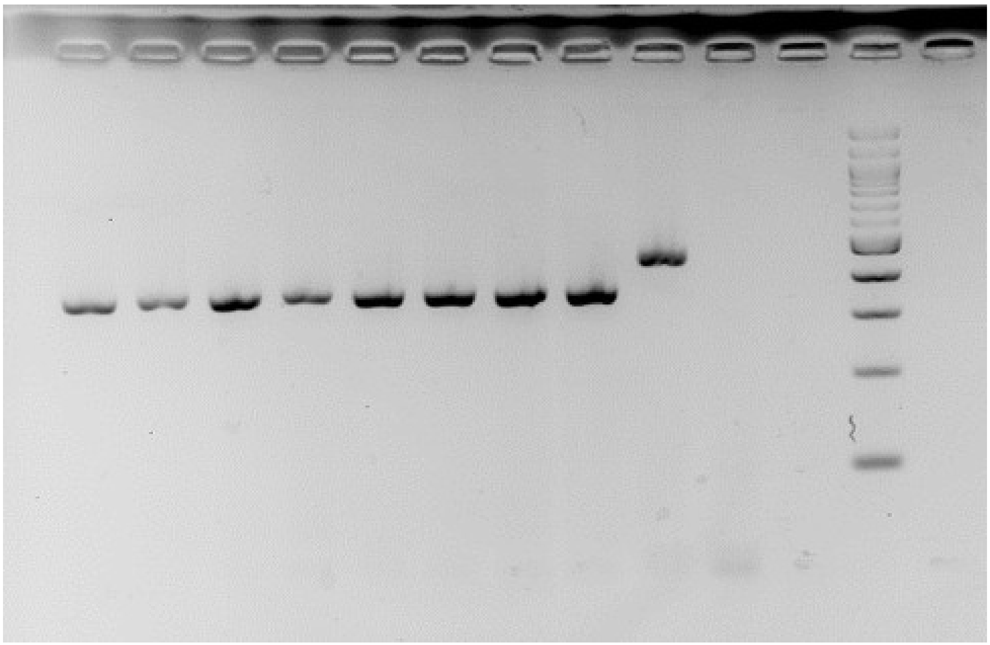

***eef1a1l1* (357 bp), Fig S5**

C C C DM DM DM L+ L- D 0 x 100 bp ladder

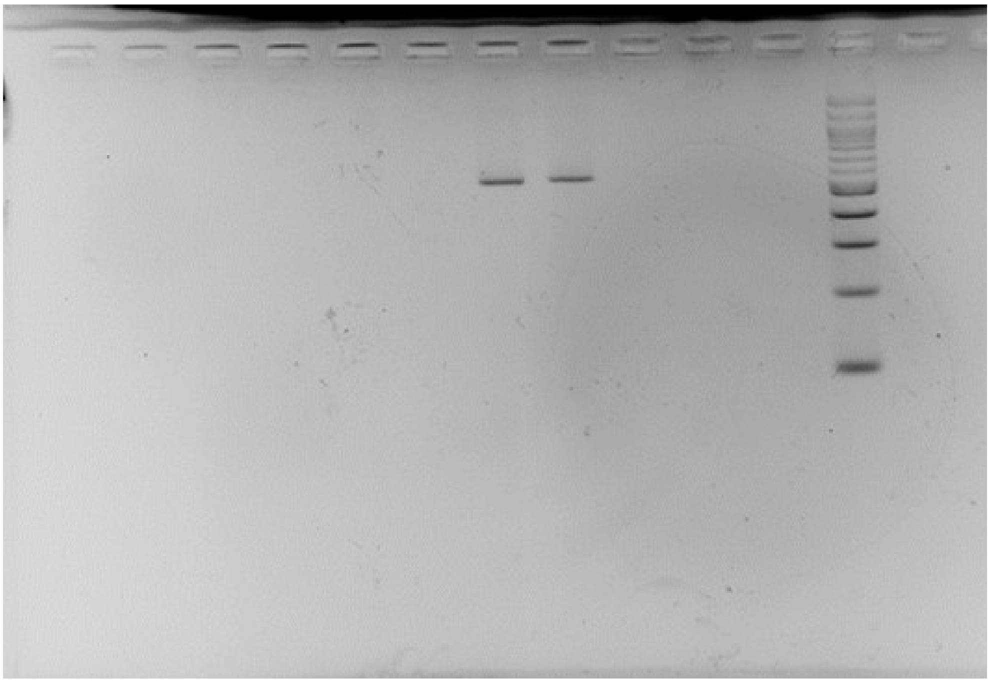

***scn1laa* (565 bp) Fig S5**

C: controls (*scn4aa*$^{+/+}$*; scn4ab*$^{+/+}$)
DM: double mutants (*scn4aa*$^{-/-}$*; scn4ab*$^{-/-}$)
L^{+} : pool of 30 6-dpf-old larvae, controls
L^{-}: pool of 30 6-dpf-old larvae, double mutants
D: genomic DNA (wild type fish, AB line)
O : negative control (water)
100 bp ladder
x : empty

BMP files, agarose gel images (EtB staining) saved in inverted colors. Pictures taken with Genesmart apparatus (real-time images from 8 bit, 768 x 582 pixel camera)

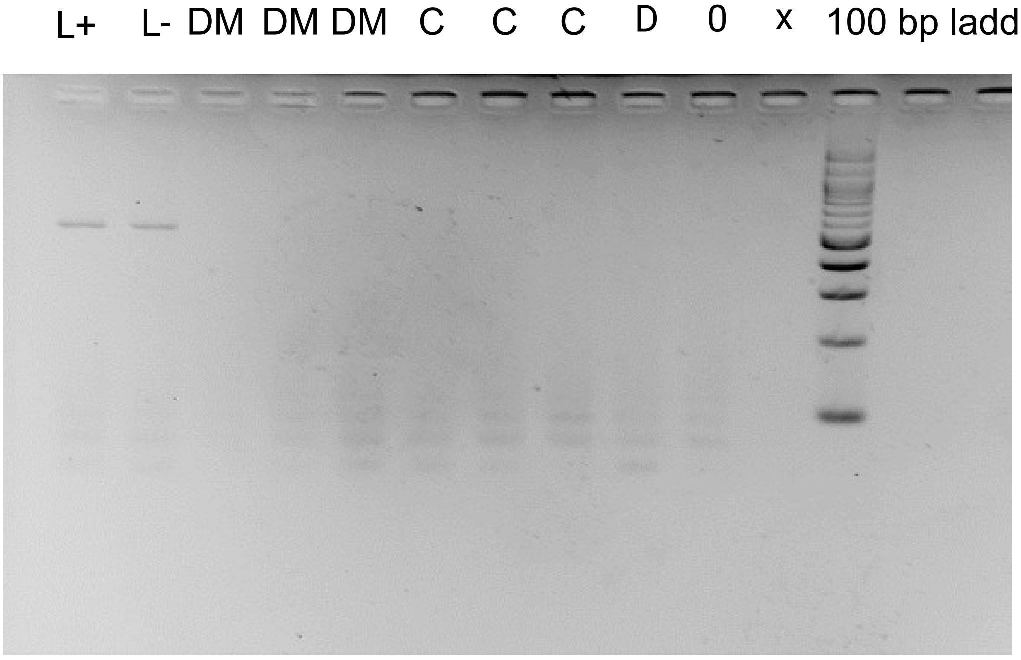

scn1lab (527 bp), Fig S5

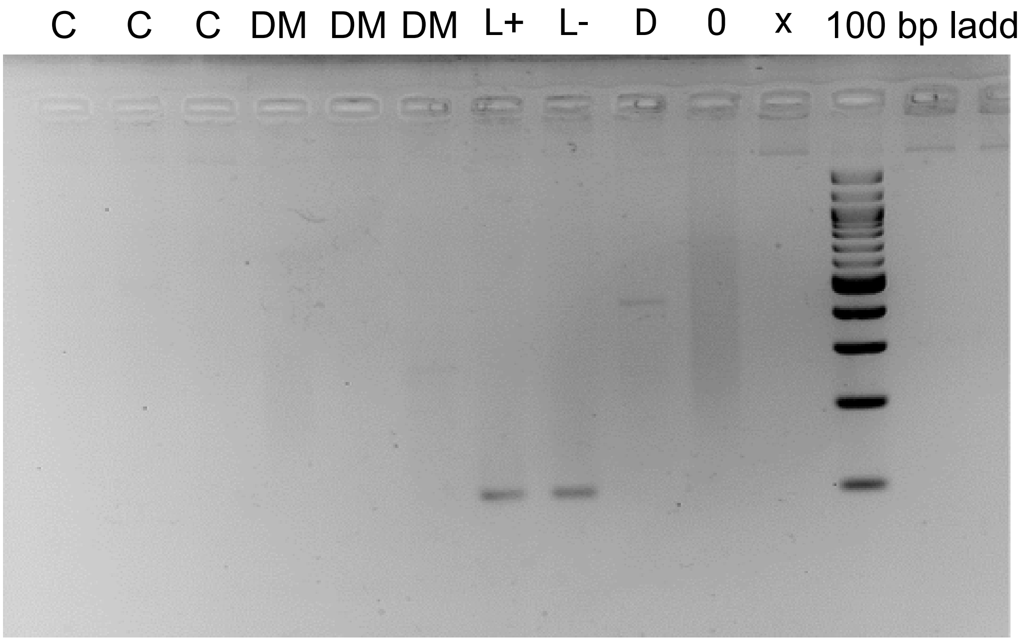

scn5laa (100 bp) Fig S5

C: controls (*scn4aa*$^{+/+}$; *scn4ab*$^{+/+}$)

DM: double mutants (*scn4aa*$^{-/-}$; *scn4ab*$^{-/-}$)

L^{+} : pool of 30 6-dpf-old larvae, controls

L^{-}: pool of 30 6-dpf-old larvae, double mutants

D: genomic DNA (wild type fish, AB line)

O : negative control (water)

100 bp ladder

x: empty

BMP files, agarose gel images (EtB staining) saved in inverted colors. Pictures taken with Genesmart apparatus (real-time images from 8 bit, 768 x 582 pixel camera)

C C C DM DM DM L+ L- D 0 x 100 bp ladder

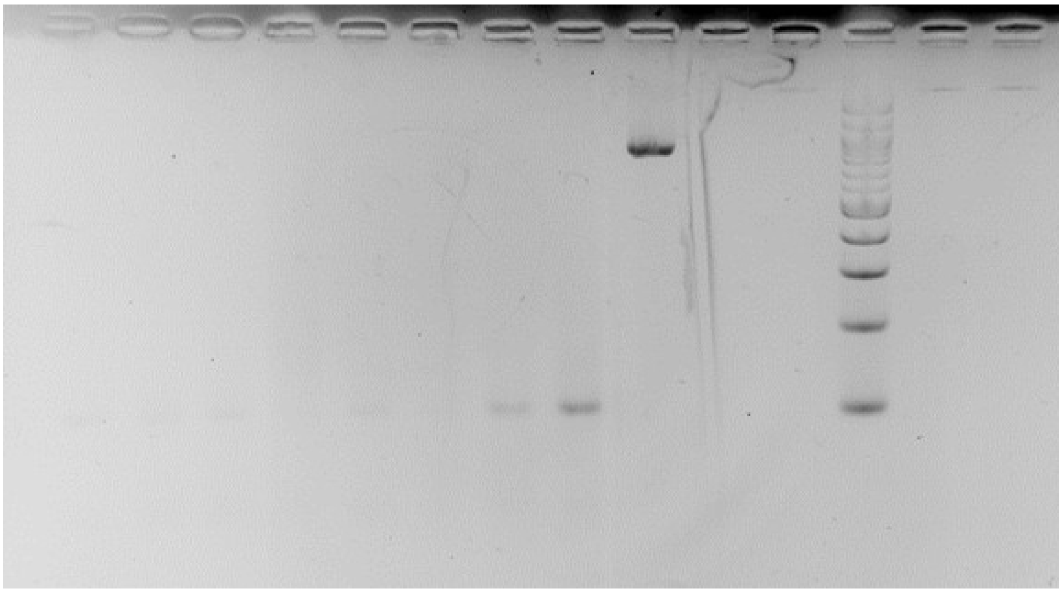

***scn5lab* (105 bp) Fig S5**

C C C DM DM DM L+ L- D 0 x 100 bp ladder

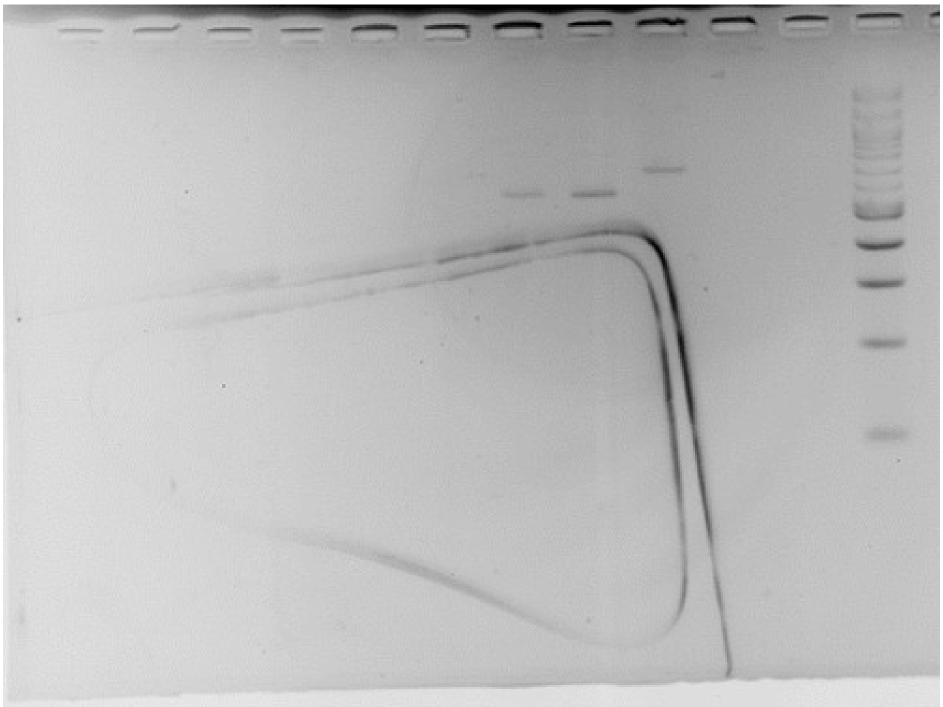

***scn8aa* (546 bp) Fig S5**

C: controls (*scn4aa*$^{+/+}$; *scn4ab*$^{+/+}$)
DM: double mutants (*scn4aa*$^{-/-}$; *scn4ab*$^{-/-}$)
L^+ : pool of 30 6-dpf-old larvae, controls
L^-: pool of 30 6-dpf-old larvae, double mutants

D: genomic DNA (wild type fish, AB line)
O : negative control (water)
100 bp ladder
x: empty

BMP files, agarose gel images (EtB staining) saved in inverted colors. Pictures taken with Genesmart apparatus (real-time images from 8 bit, 768 x 582 pixel camera)

C C C DM DM DM L+ L- D 0 x 100 bp ladder

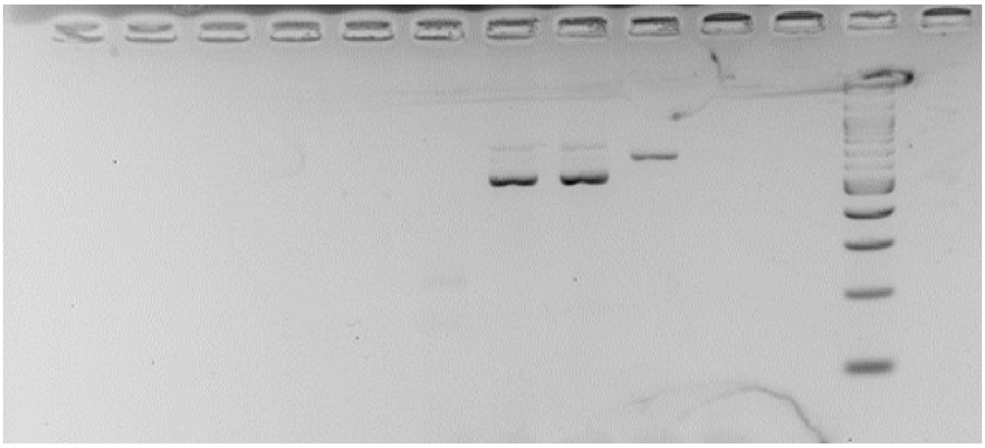

scn8ab (560 bp) Fig S5

C: controls (*scn4aa*$^{+/+}$; *scn4ab*$^{+/+}$)
DM: double mutants (*scn4aa*$^{-/-}$; *scn4ab*$^{-/-}$)
L^{+} : pool of 30 6-dpf-old larvae, controls
L^{-}: pool of 30 6-dpf-old larvae, double mutants

D: genomic DNA (wild type fish, AB line)
O : negative control (water)
100 bp ladder
x: empty
