# Supplementary material for: Zebrafish fast muscle contractions avoid the mammalian requirement for voltage-gated Na+ channels
Source: PLoS Biol. 2025 Nov 4;23(11):e3003484. doi: 10.1371/journal.pbio.3003484 (PMC12604801; doi:10.1371/journal.pbio.3003484)
Supplement: Attachment — Submitted filename: Response reviewers.pdf [file pbio.3003484.s018.pdf]

Responses to Editor and Reviewers comments

Editor comments

Thank you for your patience while your manuscript "Challenging a dogma: uselessness of voltage-gated Na^+ channels for fast muscle fibers of zebrafish to twitch" was peer-reviewed at PLOS Biology. Your manuscript has been evaluated by the PLOS Biology editors, an Academic Editor with relevant expertise, and by several independent reviewers.

As you will see in the reviewer reports, which can be found at the end of this email, although the reviewers find the work potentially interesting, they have also raised a substantial number of important concerns. Based on their specific comments and following discussion with the Academic Editor, it is clear that a substantial amount of work would be required to meet the criteria for publication in PLOS Biology. However, given our and the reviewer interest in your study, we would be open to inviting a comprehensive revision of the study that thoroughly addresses all the reviewers' comments. Given the extent of revision that would be needed, we cannot make a decision about publication until we have seen the revised manuscript and your response to the reviewers' comments. Your revised manuscript would need to be seen by the reviewers again, but please note that we would not engage them unless their main concerns have been addressed.

You'll see that the reviewers think that the study reports compelling data and would be of interest. However, none of them are convinced by the authors' characterization of the NaV channel mutant zebrafish. All reviewers recommended electrophysiological characterization of the mutant fish muscles as well as more thorough behavioral analysis. R1 and R2 pointed out the need to test for developmental compensation. R2 pointed out the need to confirm loss of functional NaV channels from the muscles. R3 also requested more careful e-phys characterization in the wild type fish muscles.

Having discussed the reviews with the Academic Editor, we emphasize the need to perform new experiments in the wild type and mutant fish. These should include electrophysiological studies describing the resting potentials and capacity for APs in the muscles, as well as characterizing the tissue to test for compensation mechanisms in the mutants. We would also strongly suggest that you carry out more thorough behavioral study of the mutants.

We greatly appreciate your invitation to submit a revised version of our manuscript.

As requested, we have performed new experiments in the wild type (WT) and mutant fish, including electrophysiological studies describing the resting membrane potentials and capacity for APs in the muscles:

- As requested, we have measured the resting membrane potential *in situ* in the conditions closest to physiological conditions (see detailed answer to point 2 of reviewer 3) in skeletal muscle fibers from WT zebrafish using intracellular microelectrodes. We found a mean value of -78 ± 0.8 mV in 124 fibers from 4 fish, a value close to the one reported by Westerfield et al. (1986) (-82 mV). We propose to illustrate this result in new Fig. S1 and to describe it page 6, line 20 from top, as follows: "Finally, we performed a series of *in situ* intracellular microelectrode recordings to measure the resting membrane potential of fast muscle fibers located in the deep region of trunk muscle using an external solution containing 2 mM K^+ (Fig. S1). The mean resting membrane potential of fast zebrafish fibers was found to be -78 ± 0.8 mV (124 fibers from 4 fish). Taken together, our data question whether APs are elicited in zebrafish fast muscle fibers in physiological conditions since, according to our data obtained on inactivation, only 20 % of Na_V are available for activation at a resting membrane potential of -78 mV." In the Discussion section, the text has been changed page 10, line 11 from top: "Second and main finding, the voltage dependence of inactivation is shifted by 25 mV toward negative voltages as compared to mouse, so that only 20 % of Na^+ channels would be available for firing APs from resting membrane potentials that we measured *in situ* (-78 mV), a value close to the one reported by Westerfield et al. (1986) (-82 mV)."

- We totally agree that evidence for absence of functional NaV in mutant was lacking in the initial version of the manuscript. We have performed new electrophysiological experiments on isolated muscle fibers from mutant fish. We found that depolarization does not elicit any voltage-gated inward current in mutant fibers, demonstrating that mutant fibers do not express functional NaV or any other voltage-gated ion channels capable of inducing depolarization. They are consequently not able to fire action potentials (APs). We propose to illustrate these new data in new Fig. 4A and describe in the Results section, page 8, line 4 from bottom: "A series of electrophysiological experiments demonstrated that depolarization pulses of increasing amplitudes elicited only voltage-gated outward currents and absolutely no inward currents in all the 27 isolated skeletal muscle fibers studied from 7 double mutant fish (Fig. 4A). These data demonstrate that NaV are not functional in fast muscles of digenic mutant fish and that no other voltage-gated channels carrying inward depolarizing currents compensate for the loss of their activity."

- In a new series of experiments performed on isolated fast muscle fibers of WT fish, we have also found that single muscle fibers, even in WT fish, do not fire APs in response to field stimulation. Indeed, we show that stimulation pulses of high voltage on isolated fibers loaded with indo-1 AM, a dye for measuring intracellular Ca^{2+}, do induce Ca^{2+} transients but that increase in size with stimulation voltage. This gives evidence that the Ca^{2+} transients are not induced by an all-or-none AP in zebrafish but result from local electrotonic responses. In line with this, Ca^{2+} transients evoked by high voltage stimulation were found to be not suppressed by exposing fibers to a Tyrode solution containing 2 µM TTX, a blocker of voltage-gated Na^{+} channels. These data demonstrate that WT zebrafish fibers do not fire AP to produce Ca^{2+} transients and to contract in response to field stimulation. In contrast, in the same experimental conditions, Ca^{2+} signals in isolated mouse muscle fibers could be elicited by stimulation of much lower voltage than zebrafish fibers and these Ca^{2+} signals were completely and reversibly suppressed by applying 2 µM TTX, proving, if necessary, that mouse muscle fibers do evoke AP-induced twitch in response to field stimulation. Together with our investigation of NaV current properties, we think that demonstrating that WT zebrafish fast muscle fibers do not fire AP in response to field stimulation in contrast to mouse fibers is a sound evidence that zebrafish fast muscle fibers are not able to fire AP, at least in our experimental conditions. Finally, considering that WT fibers do not fire AP and that NaV is absent in mutant fish fibers, we believe that it is worthless to test whether fish mutant fibers fire or not AP. We propose to illustrate these new data in new Fig. S2, S3 and S4 and to describe them in the results section as follows, page 6, line 6 from bottom: ""In order to further address this question, we tested whether isolated fast skeletal muscle fibers of zebrafish are able to twitch by firing APs in response to field stimulation. For this purpose, we measured changes in intracellular Ca^{2+} in isolated fibers loaded with the Ca^{2+} indicator indo-1 in response to 0.5 ms voltage pulses applied with field electrodes. Stimulation pulses of high voltage were found to induce a transient increase in intracellular Ca^{2+}, the size of which augmented with stimulation, and which was not affected by addition of 2 µM TTX in the external solution (Fig. S2 and S3). These data give evidence that the Ca^{2+} transients recorded in these experimental conditions result from local electrotonic responses and not from firing all-or-none AP. In parallel, we found that stimulation pulses of much lower voltage induced Ca^{2+} transients in isolated mouse muscle fibers that were completely and reversibly suppressed by addition of 2 µM TTX in the external solution (Fig. S4). These data indicate that zebrafish fast muscle fibers do not fire AP to produce Ca^{2+} transients and to contract in response to field stimulation in these experimental conditions whereas mouse muscle fibers do." In the Discussion section, the following text referring to the outcome of these experiments has been added as follows page 10, line 20: "In line with this, we found that isolated mouse muscle fibers fire APs in response to field stimulation whereas zebrafish fast fibers did not and only develop electrotonic responses insensitive to TTX."

As well as characterizing the tissue to test for compensation mechanisms in the mutants.
- Reviewers 1 and 2 ask to further characterize mutant muscles to test for compensation mechanisms. We do no dispute that some compensatory mechanisms may have occurred in the

mutant fish. However, whatever animal species, contraction of skeletal muscle fibers is always triggered by depolarization of the sarcolemma and tubular system. We have now unambiguously demonstrated that there is no voltage-gated inward current in the mutant fish, excluding any compensation by another voltage-gated channel.

Our data on WT and mutant fish highly suggest that depolarization of muscle fibers only results from opening of the acetylcholine receptors (AChRs) at the level of the neuromuscular junctions. In that context, as proposed, at least in part by the reviewers, the only possible compensation that might be envisioned in the mutant fish is strengthening of the neuromuscular transmission for alleviating the lack of voltage-gated inward depolarizing current. We have thus performed a new series of experiments on mutant fish fibers in which we labelled AChRs with alpha-bungarotoxin as we did for WT fish fibers to determine if the lack of Na^+ channels is compensated by a higher density of AChR clusters. We found that the mean and maximal distance of each point located on the extra-synaptic sarcolemma and AChR clusters were not significantly different in control and mutant fish muscle fibers. This result is important, not only by showing that the absence of Na^+ channels is not compensated by a higher density of endplates, but also by supporting our assumption that in WT fish multi-innervation enables full activation of contraction of zebrafish fast muscle fibers without the need to open voltage-gated Na^+ channels. Considering that in mono-innervated mammalian muscle fibers, opening of Na^+ channels is absolutely required for muscle fibers to contract and that knockout of the gene encoding NaV1.4 is lethal in mouse (Wu et al., 2016), the absence of motor phenotype in the mutant fish is another strong argument that NaV1.4 channels are not required for fast muscles to contract in zebrafish. These new data on mutant fish fibers have been included in the new figure 3 and the text has been changed as follows in the Results section, page 9, line 21 from top: "The absence of locomotor phenotype in mutant fish lacking functional NaV suggests that adult zebrafish does not need muscle Nav to efficiently contract and that the depolarizing signal initiating excitation-contraction coupling only results from the opening of the AChRs at the level of the multiple neuromuscular junctions. In that context, it might be envisioned that the lack of Na^+ channel depolarizing inward current in mutant muscle is compensated by potentiation of the neuromuscular transmission. In order to test this possibility, we performed AChRs labelling experiments on mutant fish as performed in WT. We found that the distribution of distances between each point located on the extra-synaptic sarcolemma and AChR clusters were very comparable in control and mutant fish. The mean and maximal distances in control (14.4 ± 0.01 µm and 58.5 ± 0.12 µm respectively) and in mutant fish (13.1 ± 0.01 and 60.6 ± 0.22 µm respectively) were not significantly different (Fig. 3C-G). This result indicates that the absence of Na^+ channels is not compensated by a higher density of endplates in mutant muscle." In the Discussion section, page 12, the mean value of maximal distance in control has been added and the percentage of attenuation of voltage changes with distance has been modified accordingly. Still in the Discussion section, the following text referring to data obtained in mutant has been added page 12, line 12 from bottom: "Along this line, our data showing that the absence of Na_V in skeletal muscle is not compensated by a higher density of endplates supports the assumption that multi-innervation of muscle fibers enables full activation of zebrafish fast muscle fibers without the need to open voltage-gated Na^+ channels."

Finally, we performed quantitative analyses of mRNAs in skeletal muscle RNA extracts from trunk skeletal muscles of adult digenic mutant fish compared to control fish. We detected no reduced levels of *scn4aa* and *scn4ab* in mutant fish and no up-regulation of any *scna* gene (6 in zebrafish) expression to compensate for the lack of functional NaV1.4a subunits. These data are illustrated in Fig. S5 and S7 and are described page 8, line 24 from top as follows: "Quantitative analyses did not detect reduced levels of *scn4aa* and *scn4ab* mRNAs in skeletal muscle RNA extracts from trunk skeletal muscles of adult digenic mutant fish compared to control fish (Fig. S7D). Up-regulation of any *scna* gene (6 in zebrafish) expression to compensate for the lack of functional $Na_V1.4a$ subunits was also not observed in mutant compared to control samples (Fig. S5)."

We would also strongly suggest that you carry out more thorough behavioral study of the mutants.

We apologize if the submitted manuscript was not clear enough on this important point. We have performed behavioral studies of the adult mutants that focus on motor responses known to depend on fast muscles contraction, *i.e.* escape responses from an aversive stimulus (see also reply to reviewer 1, points 6 and 7, and reviewer 2, point 4). In the revised version of the manuscript, we have increased the number of adult fish studied and have modified the related figure (new Fig. 4B and C) to clarify the analyses done. We have also added as supplementary materials two high-speed videos that we used to investigate the behavior of control and mutant fish showing free swimming at 30 frames per second and escape response to an aversive stimulus at 240 frames per second (videos S1 and S2). The text describing behavioral studies has been changed as follows to make it clearer, page 9, line 10 from top: "We therefore evaluated more precisely the escape response of mutant fish. Measuring the angle (head-trunk) to evaluate the head turn, did not show any difference between controls and mutants (maximal angle equal to 47.08° ± 11.79 and 59.46° ± 17.48, respectively, Fig. 4C). Latency between first escape response movement and maximal head turn was also similar between the two groups (13 ± 5 for controls and 11 ± 5 ms for mutants). The subsequent rapid acceleration was as efficient in mutants as in controls: the calculated maximal speed (157 ± 27 cm/s for controls and 161 ± 33 cm/s for mutants) and latency to attain maximal speed (8 ± 4 ms for controls and 9 ± 3 ms for mutants) were not different. Distance covered in 40 ms from the escape response start was also not altered by the lack of functional NaV in skeletal muscles (3.8 ± 0.8 cm for controls and 3.8 ± 0.9 cm for mutants).
Comparative analysis of free swimming in control and mutant fish has also been newly performed. No significant differences in swimming distance and speed have been found between the two fish populations. These data are presented in new figure S8 and the following text has been added page 9, line 3 from top: "Digenic homozygous (*scn4a*$^{-/-}$*; scn4ab*$^{-/-}$) mutants did not show altered locomotion behavior compared to homozygous (*scn4aa*$^{+/+}$*; scn4ab*$^{+/+}$) controls obtained from the same laying (20 fish per group) during usual handling (video S1 and Fig. S8)."

Additional text and figures changes in the new version of the manuscript.
- We propose to mention in the title that the study was performed in adult zebrafish. The title is now: "Challenging a dogma: uselessness of voltage-gated Na^+ channels for fast muscle fibers of adult zebrafish to twitch".

- Page 14, line 4 from top, the percentage of NaV that are not in an inactivated state at -80 mV has been changed from 26 to 20 %, according to our intracellular microelectrode data indicating a mean resting membrane potential of -78 mV.

- We omitted in the initial version of the manuscript to mention that leak currents were subtracted from recordings to measure Na^+ current amplitude. In the methods section, page 16, line 10 from top, the following sentences have been added: "Leak currents were subtracted from all recordings using the same pulse preceding every test pulse supposing a linear evolution of leak current with depolarization. After leak subtraction, the Na^+ current amplitude was determined by subtracting the current amplitude at the end of the voltage step to the current amplitude at peak."

- We propose to make slight changes in the figure (new figure 3) presenting the change in AP amplitude with resting potentials in zebrafish and in mouse. Initially, panel B presented the reduction in the mean maximal amplitude ± SEM with resting potentials, and these mean values included electrotonic responses when AP had vanished. We think that it is more informative to show the mean amplitude of spikes and only of spikes (± SD) at every resting potential and to mention the percentage of fibers that fire APs for each resting potential as illustrated below:

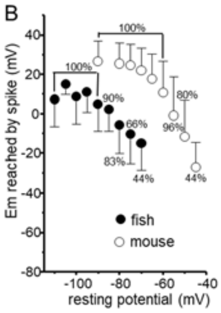

Figure 3B: Relationship between spike amplitude and resting membrane potentials in 17 fibers from 9 fish and in 25 fibers from 4 mice. Each datapoint corresponds to the mean values of voltage reached by the spike. The percentage of fibers firing APs is indicated for each resting potential. The error bars correspond to SD values that were calculated from pooled values obtained in fish and mice respectively.

- Reference to a recently published work showing that spikes can be recorded in zebrafish fast muscle at larval stage has been added in the References section and page 10, line 6 from bottom: "Shimizu et al. (2025)."

- Finally, in order to reduce the total number of figures to 4, we have combined Fig. 2 and 3 (new Fig. 2), Fig. 4 and 5 (new Fig. 3), and part of Fig. 6 and 7 in which we have integrated the figure showing the absence of voltage-gated inward currents in the mutant muscle fibers (new Fig. 4). Part of previous figure 6 has been put in supplementary data (new Fig. S7). All the figures presenting other new data, *i.e.* intracellular microelectrode recordings, absence of AP-induced twitching in isolated zebrafish fibers, quantitative PCR analyses of mRNA expression and quantitative analysis of free swimming have been added as supplementary materials (Fig. S1 to S8). Video recordings are also provided in supplementary files.

Reviewer #1

1- This manuscript has been submitted to PLoS Biology because it claims to challenge the dogma about action potentials being required for movement.

We do not really claim this assumption, which does not apply to all skeletal muscles. As it is clearly emphasized in the manuscript title, the dogma that we challenge is not that action potentials (APs) are required for movement in general but that APs are not required for movement in twitch muscle fibers of zebrafish (also named fast fibers in zebrafish or phasic fibers in mammals). Slow fibers (also named tonic fibers) are known to not fire APs since voltage-gated Na^+ currents have been reported to be absent from these muscle fibers of zebrafish.

2- The authors have previously shown that adult zebrafish muscle fibers show superfact "kinetics of action potentials" and excitation-contraction coupling. In this manuscript the authors question whether action potentials are necessary for movement. This conclusion appears to be based on a few different threads of evidence. One is that the resting potential of zebrafish muscle fibers is lower than mouse muscle fibers (although aren't resting potentials usually different between different cells?).

We do not understand this comment since we did not make any measurement of the resting membrane potential in the initial version of the manuscript. We also did not indicate somewhere in the manuscript that the resting membrane potential of zebrafish muscle fibers is lower than the resting membrane potential of mouse muscle fibers. Moreover, literature on this topic, that we refer on in our manuscript, indicates that the resting membrane potential is not different in zebrafish and in mouse muscle.

We nevertheless performed in the new version of the manuscript measurements of resting membrane potential in zebrafish muscle fibers using intracellular microelectrodes. We found a mean resting membrane potential of -78 ± 0.8 mV in 124 fibers from 4 fish (see new Fig. S1 below), a value close to the value of -82 mV reported by Westerfield et al. (1986) in the same preparation and close to the value reported in mouse muscle fibers (*i.e.* -74 mV (Dulhunty, 1978)).

We propose to add these intracellular microelectrode data to the new version of the manuscript and present the figure below as supplementary material (new Fig. S1).

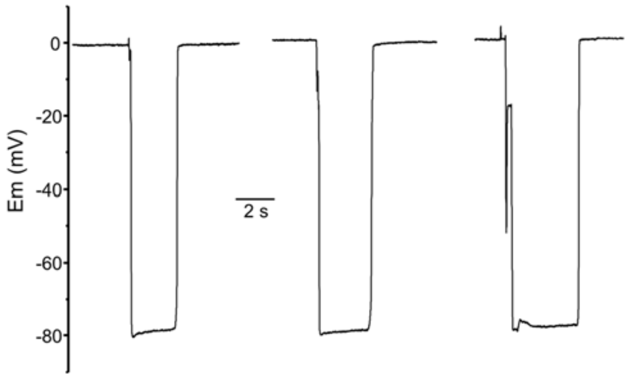

Figure S1: Resting membrane potential measured in muscle fibers using intracellular microelectrodes. Resting membrane potential was measured *in situ* in skeletal muscle fibers in euthanized WT zebrafish using intracellular microelectrodes and a micro-electrode amplifier (VF 1800, BioLogic, France) in the presence of an extracellular Tyrode solution containing 2 mM K^+ and an intra-microelectrode milieu containing 3 M KCl. The microelectrodes resistance was between 20 and 30 MΩ. Measurements were done on muscle fibers located in the deep part of the trunk accessible to microelectrodes from the interior of the fish after having removed the internal organs and a thin film lining the interior of the thorax. All the fibers are fast twitch type in this trunk region. Three different fibers were impaled with the same microelectrode that was slightly displaced laterally between each impalement. The mean resting membrane potential was found to be -78 ± 0.8 mV in 124 fibers from 4 fish.

3- It could be more informative to perturb the system and show that the two species of muscle fibers respond differently. The authors note that a sodium channel does not block movement in zebrafish (trichaine).

Slow (tonic) and fast (twitch) muscle fibers in teleosts are supposed to be activated differently, the first being activated by summation of endplate potentials from multiple neuromuscular junctions and the second proposed (until our study) to be activated by APs initiated at and propagating from multiple neuromuscular junctions since NaV current and APs could be induced in these fibers. The objective of our study was to determine if the NaV current in twitch fibers has the same properties in fish and in mammals. That is why we did not investigate the properties of slow/tonic fibers which are quasi absent from mammals (excepted a few extraocular and intrafusal muscles).
We did not write that tricaine blocks movement in zebrafish since it does. We discussed one peer-reviewed work done by another research team published in PLoS One (entitled “Anaesthetic tricaine acts preferentially on neural voltage-gated sodium channels and fails to block directly evoked muscle contraction” by Attili and Hugues, 2014), reporting that the blockade effect of tricaine at the dose recommended for fish anesthesia was resulting from a block of Na^+ channels expressed by neurons. “Although questions remain on the most appropriate agents for fish anesthesia, our study shows that tricaine at standard concentrations does not act as a paralytic neuromuscular blocking agent in zebrafish.”

4- The authors show that acetylcholine receptors are continuously distributed along the adult muscle fiber, leading the authors to hypothesize that action potential propogation may not be necessary for fiber depolarization and contraction. The authors then test this hypothesis by generating double mutants for two sodium channel genes scn4aa and acn 4ab and these mutants move normally.
It is this reviewer’s opinion that the data shown to not surpass the bar for a PLoS Biology report claiming that sodium channels/action potentials are not necessary. The data are certainly compelling, but the burden of proof for a negative result has not been met. For example, although the authors say there are two sodium channels in zebrafish it would be more correct to say there are two alpha subunits - and 14 total.
We do not well understand the point. Indeed, it is well known in the ion channel field that any voltage-gated Na^+ channel is composed of one alpha subunit that forms the pore and is sensitive to voltage for its opening and inactivation, and one or two auxiliary beta subunits that modulate the gating, kinetics and membrane expression of the alpha subunit. The alpha subunit is therefore mandatory for the Na^+ channel to function as a channel, not the beta subunit. In zebrafish, 8 *scna* genes encode alpha subunits of voltage-gated Na^+ channels, and 5 genes encode auxiliary beta subunits. We did not write that there are two sodium channels in zebrafish. We reported from the literature that only 2 of the 8 *scna* genes encoding a pore forming subunit are expressed in skeletal muscles of adult zebrafish. These two genes are *scn4aa* and *scn4ab*. We confirmed this critical gene expression point by PCR analyses on control samples (new Fig. S5 below). We also showed in the revised version of the manuscript the absence of gene expression upregulation of another *scna* gene in muscle samples of mutants to compensate for the loss-of-function mutations in the two *scn4a* genes. We now more clearly specify in the Discussion section that the phylogenetic and synteny analyses have been performed on *scna* genes and not all *scn* genes.

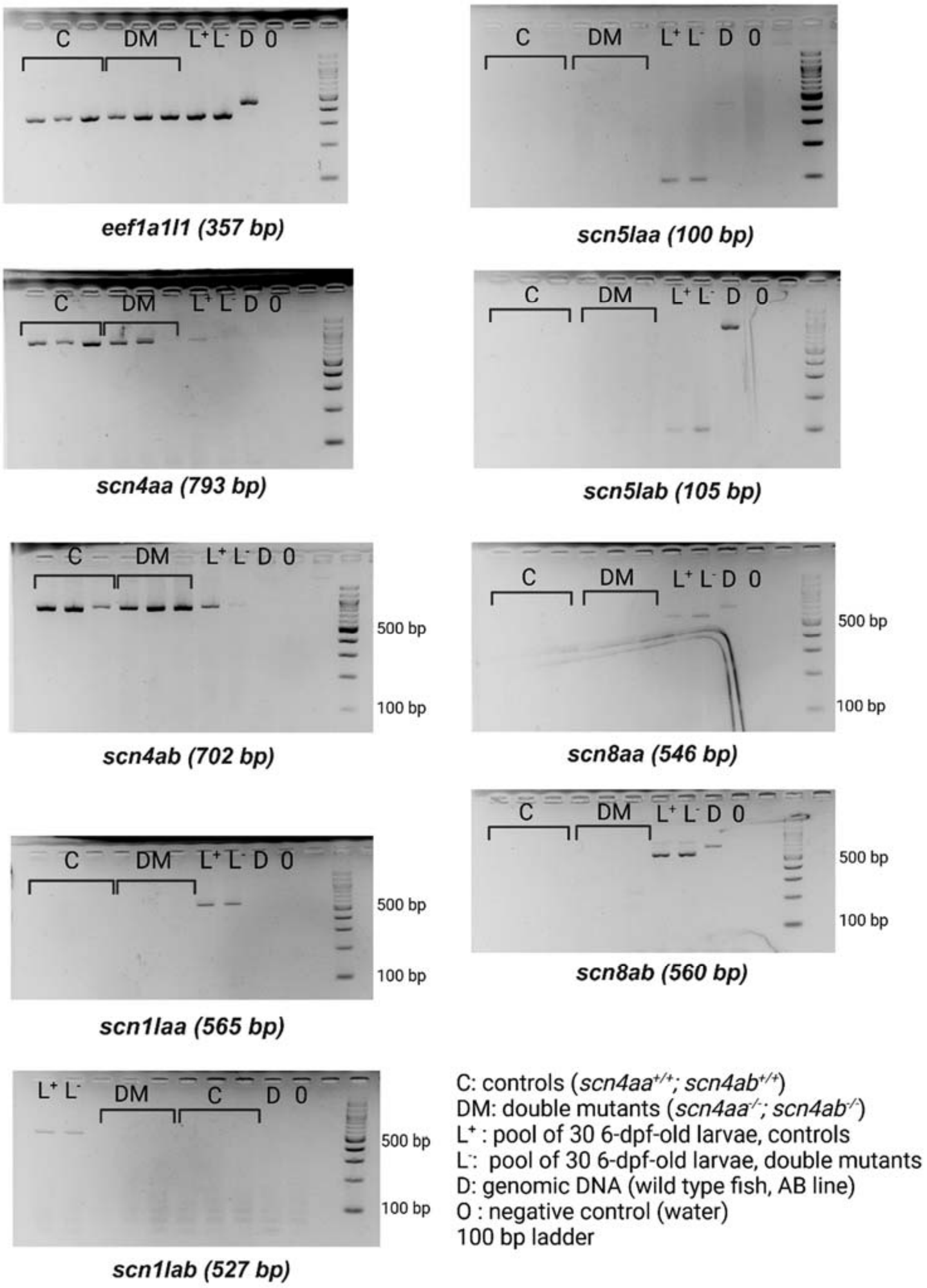

Figure S5: PCR analysis of gene expression of *scna* genes. PCR amplification was done using cDNA samples obtained by reverse-transcription of total RNA samples extracted from trunk muscles of control (C) and digenic *scn4aa*$^{-/-}$;*scn4ab*$^{-/-}$ mutant (DM) adult fish (3 fish by genotype) and control (L+) and digenic mutant (L-) 6-days-post fertilization(dpf) larvae (pool of 30 larvae). Genomic DNA (D, 25 ng) was used to demonstrate the absence of genomic DNA contamination in cDNA samples, and water (0) to demonstrate the absence of any PCR contamination. The eef1a1l1 gene was used as control of reverse-transcription efficiency. Primers used are listed in Table S2. A touch-down protocol was used for PCR amplification. A single amplicon at the expected size was obtained for all genes in the larval samples as expected since whole larvae were used for RNA extraction. In trunk muscle extracts of control and double mutant samples, PCR amplicons were only obtained for scn4aa and scn4ab, confirming that *scn4a* are the sole *scna* isoforms expressed in control muscles of zebrafish and suggesting the lack of upregulation of another *scna* gene expression in muscles of double mutant fish.

Additionally, we are surprised that the reviewer mentions that “the burden of proof for a negative result has not been met” without mentioning all the electrophysiological work done on WT fish and

comparison made with mouse muscle: our work shows that (i) 80 % of NaV are inactivated at the mean resting membrane potential (-78 mV) that we measured in the new set of experiments, (ii) APs cannot be elicited at this mean resting potential under current clamp conditions, (iii) fast muscle fibers are multi-innervated with such a density of AChRs that there is no need to propagate APs when considering the negligible attenuation of endplate potentials traveling along the muscle cell membrane *via* passive electrical conduction for a 2 mm skeletal muscle length constant (λ).

5- Without an understanding of the impacts of the other subunits on potential compensatory activity, without understanding the impact of the double mutants on muscle physiology and muscle structure, it seems like it is premature to claim that these are not necessary.
We agree with the reviewer that compensatory mechanisms may have resulted from the knockout of the genes encoding muscle voltage-gated Na^+ channels. We have performed new experiments in fibers isolated from the double mutant fish to better investigate their muscle physiology as suggested. We found that voltage pulses of increasing amplitudes applied from a holding potential of -110 mV do not elicit any voltage-gated inward depolarizing current but only voltage-gated outward currents that were inhibited by TEA and replacement of external Cl^- by $MeSO_3$ (see new Fig. 4A below). Inward currents were never detected in all the 27 fibers from 7 double mutant fish studied, demonstrating that NaV are not functional in mutant muscles. This is a functional demonstration that no other pore-forming Na^+ channel alpha subunit or voltage-gated channel carrying inward current compensate for the absence of NaV. Thus, if NaV is necessary, we do not see how a change in muscle physiology or muscle structure could compensate for the lack of membrane depolarization due to the absence of functional NaV, except in neuromuscular transmission that we investigated (see answer to the last point below).
We propose to add the following voltage clamp data obtained on the double mutant to the new version of the manuscript as new figure 4A.

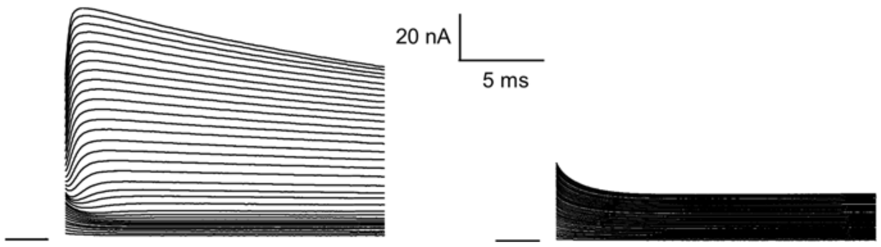

Figure 4A: Absence of voltage-gated inward currents in isolated fast skeletal muscle fibers of the double mutant zebrafish. Left panel shows currents evoked by depolarizing pulses of increasing amplitudes (steps of 5 mV) from a holding potential of -110 mV applied from -100 to +40 mV in the presence of an external Tyrode solution. Right panel shows the effect of an external solution containing 20 mM TEA and in which external Cl^- has been replaced by $MeSO_3$ on currents elicited by the same voltage protocol in the same fiber.

6- Is it at all possible that muscle fibers incorporate more slow characteristics, and perhaps acceleration is impacted whereas the average speed is not? The data shown are all in the first 32ms following the stimulus. Why was this time frame chosen?
As underlined by the reviewer, our expectation was that Nav1.4 knock-out results in the specific loss of ultrafast swim and not the slow/intermediate swim. Two main swim behaviors are recognized in zebrafish: the C bend (a single powerful stereotypic contraction), which mediates escape, and rhythmic swim. Fast muscles are reported to be required for ultrafast swim. For example, knocking-out the AChR epsilon subunit, which is specifically expressed by fast fibers, results in a reduced “first turn angle” (C bend) in the few mutants obtained at the adult age (25% of the homozygous mutants) compared to WT fish but no change in the swim speed (Zempo et al., 2020, Sci Adv 6:eaax8382). Our expectation was therefore that Nav1.4 knock-out results in the loss of ultrafast swim and not slow/intermediate swim. We performed 1 s-long high-speed video (240 frames per second (fps)) to

record the escape response swim in response to an aversive element in control and mutant fish (Supplementary video S2). In our assay, the first turn of the escape response and the higher swim speed were observed within 20 ms after the repulsive stimulus (see new figure 4B and C below). We therefore chose 40 ms as time frame in order to best focus our analyses on ultrafast swim. We first analyzed the average speed as noticed by the reviewer, but also the first turn angle (C-bend shape by measuring the angle between the head and the trunk), and the ultrafast swim speed (calculated using the distance traveled by each fish head between two adjacent frames (bin of 4 ms)). We replaced the average speed by the measurement of the total distance to not mask any subtle difference in speed, and add the latency analyses (minimal first turn angle and maximal speed). We are thus confident that swimming behavior supported by fast muscles is not affected by the lack of inward current and AP in myofibers of mutant fish.

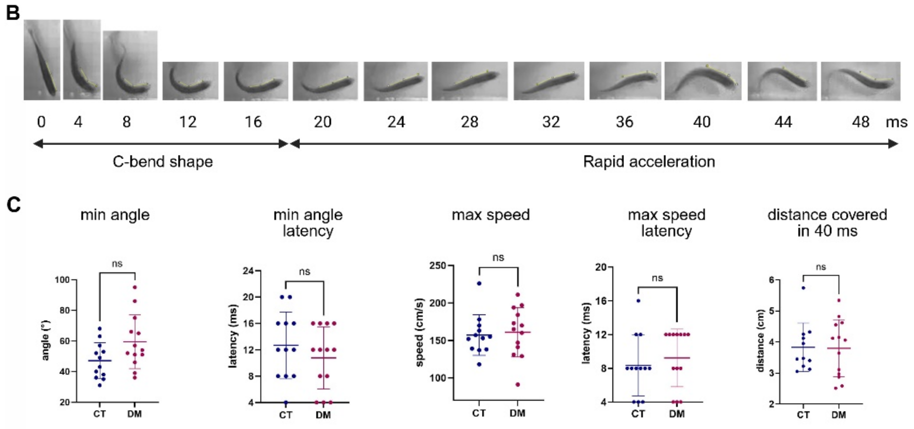

Figure 4B and C: Phenotype of *scn4aa*$^{-/-}$; *scn4ab*$^{-/-}$ fish. (B) Example of swim escape response of one mutant (*scn4aa*$^{-/-}$; *scn4ab*$^{-/-}$) fish. Images were extracted from the high-speed video (one frame each 4 ms). The C-bend shape occurs between the first 16 ms followed by an ultrafast swim. (C) Quantitative analyses of C-bend shape (minimal head-trunk angle and time to reach minimal angle) and following swim (maximal speed, maximal speed latency and total distance covered in 40 ms following the escape response start) calculated for controls (blue) and mutants (red). No difference statistically significant was seen between the two groups whatever the parameter studied. Four- to twelve-month-old fish were studied (minimum of 11 per genotype). Graphs represent mean +/- SD with individual values (unpaired Mann-Whitney tests).

7- What about normal motility tests that analyze dark/light average swim speeds/velocity/acceleration over longer time frames?
The white light-induced tests over long period of time suggested by the reviewer are usually performed on larvae (from 4 dpf to 7 dpf) in order to explore their sensory-motor response induced by abrupt alternance of light and dark. Larvae are not the scope of this work, which focuses on adults. In addition, the camera speed that can be used for recording video on a long time is low (usually 30 fps). Based on the existing literature, it is our opinion that these motility tests cannot accurately investigate the ultra and fast swim of adult zebrafish. In the revised version, we have completed the behavioral description of the adult mutants by analyzing their free swim at 30 fps during 10 s. No difference in average and maximal speed with controls was observed. These data have been added as supplementary video 1 and Fig. S8 presenting quantitative analysis of free swimming in WT and mutant fish.

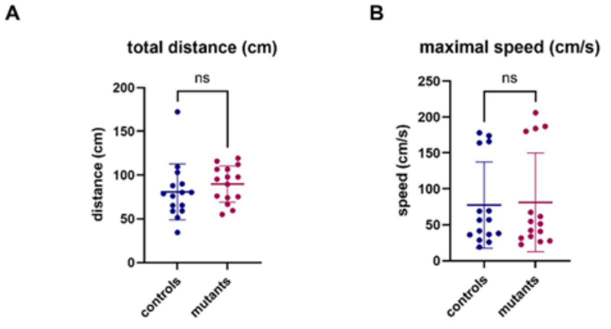

Figure S8: Quantitative analyses of free swimming in WT and mutant fish. (A) Total distance covered in 10 s. (B) Maximal speed. Graphs represent mean ± SD with individual values (unpaired Mann-Whitney test) (4 to 12-month-old, minimum of 10 fish per genotype).

8- Is it possible that the escape response does not need these subunits, and other subunits/muscle types can compensate during general swimming?
As mentioned above, our new electrophysiological data on the double mutant indicate that depolarization does not activate any inward current. Together with the behavioral analyses, we conclude from these experiments that neither the escape response, nor general swimming involve any other voltage-gated channel subunit than the NaV1.4 alpha subunits. The hypothesis according to which other subunits may have compensated the absence of the alpha subunits during swimming can thus be ruled out.

9- Is it known in other systems what the impact of deleting the alpha subunit is?
As emphasized in the manuscript, deleting the voltage-gated Na^+ channel alpha subunit in mammalian skeletal muscle is lethal, since, in contrast to zebrafish, mammalian skeletal muscles are unable to contract in the absence of AP. This emphasizes the significance of our study.

10- How is resting potential altered? How is Ca^{2+} signaling altered? Is there increased innervation, are there more AChR receptors per fiber in the double mutants? These questions are ones that could help the authors present a more compelling story.
We did not study the resting potential and Ca^{2+} signaling in double mutants, since any change occurring on these parameters, cannot physiologically compensate for the absence of voltage-activated inward depolarizing current on membrane depolarization and excitation-contraction coupling. As mentioned above, we do not dispute that compensatory mechanisms may have occurred in the double mutant. However, whatever animal species, contraction of skeletal muscle fibers is always triggered by depolarization of the sarcolemma and tubular system. We have now unambiguously demonstrated that there is no voltage-gated inward current in the muscle fibers of double mutant fish, giving evidence that depolarization of muscle fibers can only result from opening of the AChRs at the level of the neuromuscular junctions as observed in tonic fibers. In that context, the only possible compensation that might be envisioned in the mutant fish is potentiation of the neuromuscular transmission for alleviating the lack of Na^+ channel depolarizing inward current. We have thus performed a new series of experiments on mutant muscle fibers in which we labelled AChRs with alpha-bungarotoxin as we performed in WT fish to determine if the lack of Na^+ channels is compensated by a higher density of endplates. As shown in new figure 3 below, we found that the mean and maximal distance of each point located on the extra-synaptic sarcolemma and AChR clusters were not significantly different between WT and mutant fish. This result is important, since it shows that the absence of Na^+ channels is not compensated by a higher density of endplates. It also supports the assumption that multi-innervation of muscle fibers enables full activation of zebrafish fast muscle fibers without the need to open voltage-gated Na^+ channels in WT fish. Considering that in mono-innervated mammalian muscle fibers, opening of Na^+ channels is absolutely required for muscle fibers to contract and that knockout of the gene encoding the pore forming subunit of NaV1.4 is lethal (Wu et al., 2016), the absence of phenotype in the double mutant can be again taken as a strong argument to demonstrate that NaV and AP are not required for fast muscle to contract in zebrafish. We propose to include these data in the new version of the manuscript as a new panel in figure 3, added to the panels presenting data on WT fibers.

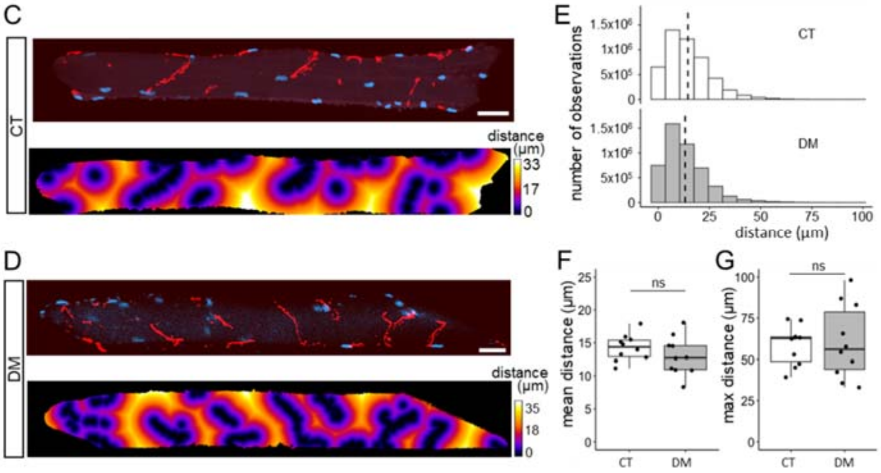

Figure 3C-G: Distance analysis of AChRs clusters on isolated muscle fibers from control and *scn4aa*$^{-/-}$*;scn4ab*$^{-/-}$ mutant zebrafish. (C, D) AChRs and nuclei labelling with α-bungarotoxin (red) and Hoechst (blue) respectively in fast muscle fibers from control (C, *CT*) and mutant fish (D, *DM*). Measurements were made on Z-stacks images of whole fibers by dividing each fiber at its equator and applying Z-projection to each half. Z-projections of the upper half of a representative CT and DM fiber are shown (upper panels). Euclidean distance transformation of half-fiber Z-projection generated a distance mapping visualized by colored gradient (lower panels). Bar = 30 µm. (E) Euclidian distances corresponding, for each point of the extra-synaptic sarcolemma, to the shortest distances to the nearest AChRs clusters were collected and histogram of the distribution of pooled data of 10 fibers from 3 control (top) and 10 fibers from 4 mutant fish (bottom) is shown. The dash lines correspond to mean values. (F) Box plots of mean distance in control (*CT*) and in mutant (*DM*) fibers. (G) Box plots of maximal distance in control (*CT*) and mutant (*DM*) fibers. Wilcoxon test shows the absence of significative difference for mean and maximal distances between mutant and control (P = 0.22 and P = 1.0, respectively). Data points of each fiber are shown by filled circles.

Reviewer #2

This study challenges the established role of voltage-gated sodium channels (NaV) in zebrafish fast muscle fibers by presenting electrophysiological and genetic evidence suggesting that these channels are not essential for muscle contraction. The topic is interesting and has implications for our understanding of muscle physiology and neuromuscular diseases. However, while the study presents robust electrophysiological data, several significant weaknesses limit its impact. In particular, the analysis of NaV mutants is incomplete and lacks crucial complementary approaches such as immunohistochemistry and further electrophysiological characterization. Below, I detail my concerns and suggest necessary revisions to improve the manuscript.

1- The study generates double knock-out (*scn4aa-/-*, *scn4ab-/-*) zebrafish but does not provide adequate validation of the loss of NaV channels at the protein level. The absence of functional NaV in these mutants is inferred but not directly demonstrated. Immunohistochemistry should be performed to confirm the complete loss of NaV protein in muscle fibers. Western blot analysis or quantitative PCR showing mRNA non-sense mediated decay could further substantiate the absence of NaV expression in skeletal muscle.

It is true that in the initial version of the manuscript, we did not directly demonstrate the absence of functional NaV in muscle fibers of *scn4a* double mutant fish. We performed quantitative PCR analyses of *scn4aa* and *scn4ab* gene expression in skeletal muscle samples of adult fish and did not observe any evidence of reduced level suggesting nonsense mediated decay. This result is now illustrated in new Fig. S3. This is rather good news since this regulatory mechanism has been shown to be a signal for gene compensation in zebrafish (El-Brolosy et al., 2019). Immunostaining NaV channels in muscle myofibers is a very challenging task in mammals, further complicated in zebrafish by the non-conservation of antibody epitopes. This is why we could not show immunostaining or western-blot data.

To undoubtedly demonstrate the functional NaV knock-out in the muscle fibers of *scn4a* double mutants, we have performed a new set of electrophysiological experiments on muscle fibers from the double mutant fish. Our new data, illustrated in Fig. 4A below, demonstrate that depolarization does not activate any inward current in fast muscle fibers from the double mutant fish. We believe that this result is the strongest evidence possible to demonstrate the complete loss of functional NaV in fast muscle fibers of the double mutant. We propose to add these data to the new version of the manuscript as new figure 4A.

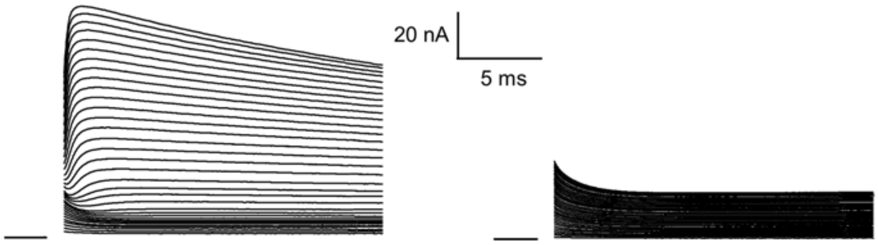

Figure 4A: Absence of voltage-gated inward currents in isolated fast muscle skeletal muscle fibers of the double mutant zebrafish. Left panel shows currents evoked by depolarizing pulses of increasing amplitudes (steps of 5 mV) from a holding potential of -110 mV applied from -100 to +40 mV in the presence of an external Tyrode solution. Right panel shows the effect of an external solution containing 20 mM TEA and in which external Cl- has been replaced by $MeSO_3$ on currents elicited by the same voltage protocol in the same fiber.

2- Skeletal muscle could have plasticity in response to genetic perturbations. The study does not examine whether alternative mechanisms compensate for the loss of NaV, such as changes in ion channel expression or synaptic architecture. A transcriptomic or proteomic analysis would help determine whether other ion channels are upregulated in response to NaV loss. Electrophysiological studies of synaptic inputs, such as miniature endplate potentials (mEPPs) or spontaneous synaptic currents, would provide insights into neuromuscular transmission in mutants.

It is totally valid to hypothesize that compensatory mechanisms may have occurred in the double mutant. However, whatever animal species, contraction of skeletal muscle fibers is always triggered

by depolarization of the sarcolemma and tubular system. We have now unambiguously demonstrated that there is no voltage-gated inward current in the muscle fibers of mutant fish, giving evidence that depolarization of muscle fibers can only result from opening of the AChRs at the level of the neuromuscular junctions as observed in tonic fibers.

Regarding the possible compensation by changes in synaptic transmission, the very low maximal distance that we calculated between extrasynaptic sarcolemma and any AChR cluster on WT muscle fibers as compared to the length constant (λ) highly suggests that the simultaneous recruitment of endplates would be sufficient to induce excitation-contraction coupling without the need of AP propagation. If we did not record spontaneous synaptic event as suggested by the reviewer, we performed a new series of experiments on mutant fish in which we labelled AChRs with alpha-bungarotoxin as we performed in WT fish to determine if the lack of Na$^+$ channels might be compensated by a higher density of endplates. As shown in Fig. S8 below, we found that the mean and maximal distance of each point located on the extra-synaptic sarcolemma and AChR clusters were not significantly different between WT and mutant fish. This result is important, since it shows that the absence of Na$^+$ channels is not compensated by a higher density of endplates. It also supports the assumption that multi-innervation of muscle fibers enables full activation of zebrafish fast muscle fibers without the need to open voltage-gated Na$^+$ channels in WT fish. Considering that in mono-innervated mammalian muscle fibers, opening of Na$^+$ channels is absolutely required for muscle fibers to contract and that knockout of the gene encoding the pore forming subunit of NaV1.4 is lethal (Wu et al., 2016), the absence of phenotype in the double mutant can be again taken as a strong argument to demonstrate that NaV and APs are not required for fast muscle to contract in zebrafish. We propose to include these data in the new version of the manuscript as a new panel in figure 3, added to the panels presenting data on WT fibers.

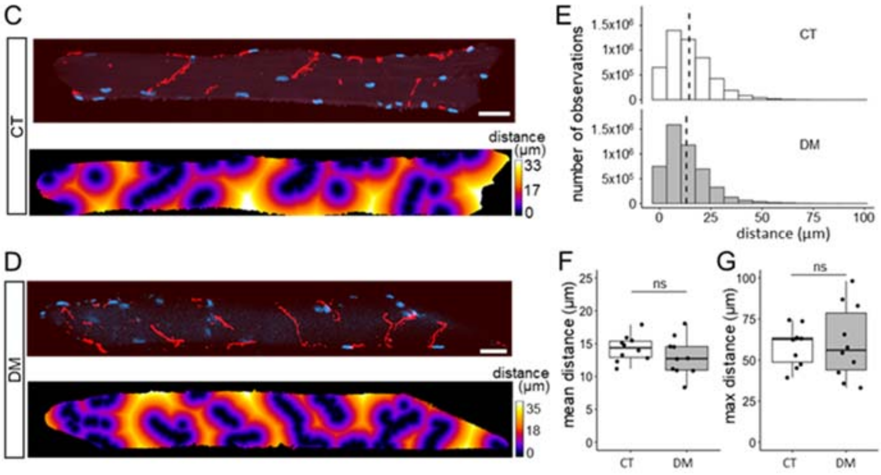

Figure 3C-G: Distance analysis of AChRs clusters on isolated muscle fibers from control and *scn4aa*$^{-/-}$;*scn4ab*$^{-/-}$ mutant zebrafish. (C, D) AChRs and nuclei labelling with α-bungarotoxin (red) and Hoechst (blue) respectively in fast muscle fibers from control (C, *CT*) and mutant fish (D, *DM*). Measurements were made on Z-stacks images of whole fibers by dividing each fiber at its equator and applying Z-projection to each half. Z-projections of the upper half of a representative CT and DM fiber are shown (upper panels). Euclidean distance transformation of half-fiber Z-projection generated a distance mapping visualized by colored gradient (lower panels). Bar = 30 µm. (E) Euclidian distances corresponding, for each point of the extra-synaptic sarcolemma, to the shortest distances to the nearest AChRs clusters were collected and histogram of the distribution of pooled data of 10 fibers from 3 control (top) and 10 fibers from 4 mutant fish (bottom) is shown. The dash lines correspond to mean values. (F) Box plots of mean distance in control (*CT*) and in mutant (*DM*) fibers. (G) Box plots of maximal distance in control (*CT*) and mutant (*DM*) fibers. Wilcoxon test shows the absence of significative difference for mean and maximal distances between mutant and control (P = 0.22 and P = 1.0, respectively). Data points of each fiber are shown by filled circles.

3- The authors claim that NaV deletion does not affect locomotion but provide only qualitative observations. More quantitative locomotion assays, such as high-speed videography with kinematic analysis, should be conducted.

We are surprised by the comment since we performed quantitative and not qualitative analyses. Two main swim behaviors are recognized in zebrafish: the C bend (a single powerful stereotypic contraction), which mediates escape, and rhythmic swim. Fast muscles are reported to be required for ultrafast swim. For example, knocking-out the AChR epsilon subunit, which is specifically expressed by fast fibers, results in a reduced "first turn angle" (C bend) in the few mutants obtained at the adult age (25% of the homozygous mutants) compared to WT fish but no change in the swim speed (Zempo et al., 2020, Sci Adv 6:eaax8382). Our expectation was therefore that Nav1.4 knock-out results in the loss of ultrafast swim. We performed high-speed video (1 s long, 240 frames per second (fps)) to record the escape response swim of control and mutant fish in response to a repulsive element (Supplementary video S2). In our assay, the first turn of the escape response and the higher swim speed were observed within 20 ms after the repulsive stimulus (see new Fig. 4 below). We therefore chose 40 ms as time frame in order to best focus our analyses on ultrafast swim. We calculated the first turn angle (C-bend shape by measuring the angle between the head and the trunk), the ultrafast swim speed (calculated using the distance traveled by each fish head between two adjacent frames (bin of 4 ms)) and the total distance traveled during 40 ms. We added in the revised version the latency between movement initiation and minimal angle and maximal speed. We modified the corresponding figure 4 to clarify our behavioral analyses in the revised version.

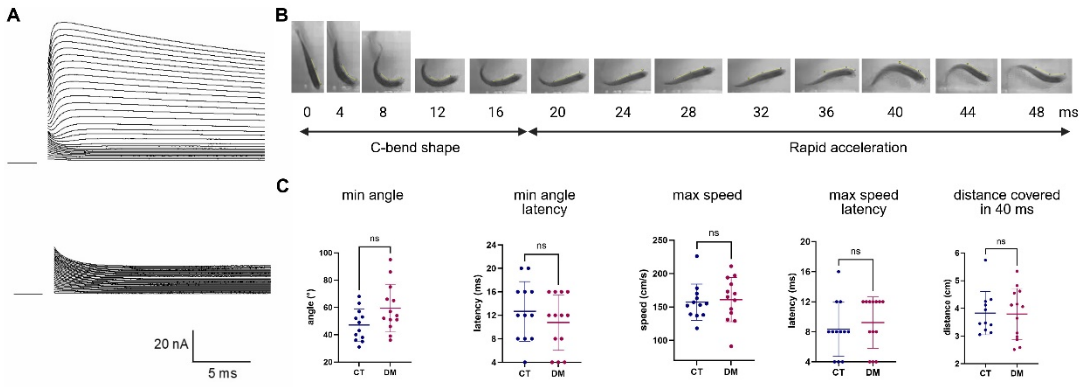

Figure 4: Phenotype of *scn4aa*$^{-/-}$; *scn4ab*$^{-/-}$ fish. (A) Absence of voltage-gated inward currents in isolated fast muscle skeletal muscle fibers of the double mutant zebrafish**.** Upper panel shows currents evoked by depolarizing pulses of increasing amplitudes (steps of 5 mV) from a holding potential of -110 mV applied from -100 to +40 mV in the presence of an external Tyrode solution in a mutant fiber. Lower panel shows the effect of an external solution containing 20 mM TEA and in which external Cl^- has been replaced by $MeSO_3^-$ on currents elicited by the same voltage protocol in the same fiber. (B). Example of swim escape response of one mutant (*scn4aa*$^{-/-}$; *scn4ab*$^{-/-}$) fish. Images were extracted from the high-speed video (one frame each 4 ms). The C-bend shape occurs between the first 16 ms followed by an ultrafast swim. (C) Quantitative analyses of C-bend shape (minimal head-trunk angle and time to reach minimal angle) and following swim (maximal speed, maximal speed latency and total distance covered in 40 ms following the escape response start) calculated for controls (blue) and mutants (red). No difference statistically significant was seen between the two groups whatever the parameter studied. Four- to twelve-month-old fish were studied (minimum of 11 per genotype). Graphs represent mean +/- SD with individual values (unpaired Mann-Whitney tests).

We also completed the behavioral description of the adult mutants by analyzing the free swim at 30 fps (10 s). No difference in average and maximal speed with controls was observed between controls and mutants (Supplementary Video 1 and new Fig. S8). We are thus confident that swimming behavior supported by fast muscles is not affected by the lack of inward current and AP in myofibers of mutant fish.

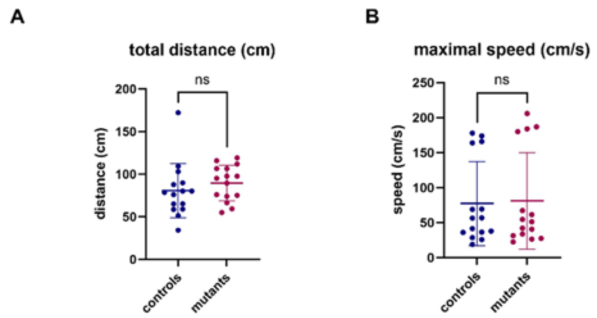

Figure S8: Quantitative analyses of free swimming in WT and mutant fish. (A) Total distance covered in 10 s. (B) Maximal speed. Graphs represent mean ± SD with individual values (unpaired Mann-Whitney test) (4 to 12-month-old, minimum of 10 fish per genotype).

4- Overall, the conclusion that zebrafish fast muscle fibers do not require NaV for contraction is intriguing but may be overstated. A possibility not considered is that NaV loss might lead to developmental compensation that is not present in wild-type fish. An analysis of neuromuscular junction development by immunohistochemisty in mutant and WT should also be performed.

As presented above (point 2), we have performed labelling experiments on WT (already shown in the initial version of the manuscript) and in double mutant fish and found that there was no change in the density of endplates in double mutant as compared to WT fish.

Reviewer #3

In this manuscript the authors present data to argue that skeletal muscle voltage gated Na channels (NaV1.4) do not perform the typical function associated with these channels in adult zebrafish fast twitch skeletal muscle fibers. The authors use a variety of techniques including electrophysiology, imaging, molecular and behavioral tools to show that adult zebrafish fast twitch fibers produce APs that peak at a lower voltage than mouse fast twitch fibers, are shifted towards more negative voltages in their activation and inactivation kinetics, and cannot elicit robust APs at resting potentials more depolarized than around -80 mV. The authors find that synaptic terminals and endplate contacts are located in close proximity and that genetic manipulation leading to inactive or non-functional NaV1.4 channels does not impact swimming in adult fish.

The work is well done and provides a reasonable argument to support the author's conclusions. The comparison with mouse fibers is compelling and is an important aspect of the study. It provides a control for the techniques and suggests that the experimental protocols are well founded. I have a few comments below but my main concern relates to confirming the resting potential of these fibers in adult animals (or as close to in situ as possible), and voltage and current clamping mutant fibers as mentioned below.

Comments are listed below.

1. I am surprised that the voltage threshold for Na^+ current activation is significantly different between the mouse (-65 +/- 6 mV) and zebrafish (-59 +/- 7 mV). Please double check.
We have double checked the respective values and the threshold for Na^+ current activation is indeed higher in zebrafish (-59 mV) as compared to mouse (-65 mV). It looks like not only voltage-dependence of inactivation but also voltage-dependence of activation is different in mouse and zebrafish.

2. A determination of the resting membrane potential in adult zebrafish fast twitch fibers would be beneficial to the study. What is the true resting membrane potential of these fibers in situ?
As requested by the reviewer, we measured *in situ* the resting membrane potential in skeletal muscle fibers in euthanized WT zebrafish using intracellular microelectrodes and a microelectrode amplifier in the presence of an extracellular Tyrode solution containing 2 mM K^+ and an intra-microelectrode milieu containing 3 M KCl. The microelectrodes resistance was between 20 and 30 MΩ. Measurements were done on muscle fibers located in the deep part of the trunk accessible to microelectrodes from the interior of the fish after having removed the internal organs and a thin film lining the interior of the thorax. All the fibers are fast twitch type in this trunk region. Three different fibers were impaled with the same microelectrode that was slightly displaced laterally between each impalement (Fig. S1 below). We found a mean resting membrane potential of -78 ± 0.8 mV in 124 fibers from 4 fish. This value is very close to the value of -82 mV found by Westerfield et al. (1986). We propose to add these intracellular microelectrode data to the new version of the manuscript as new figure S1.

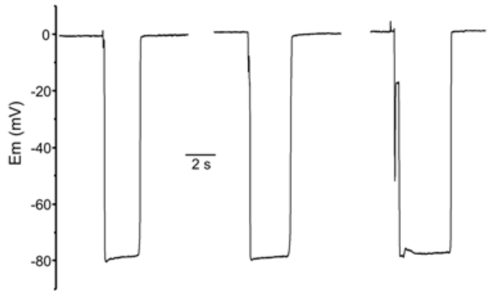

Figure S1: Resting membrane potential measured in muscle fibers using intracellular microelectrodes. Resting membrane potential was measured *in situ* in skeletal muscle fibers in euthanized WT zebrafish using intracellular microelectrodes and a micro-electrode amplifier (VF 1800, BioLogic, France) in the presence of an extracellular Tyrode solution containing 2 mM

K^+ and an intra-microelectrode milieu containing 3 M KCl. The microelectrodes resistance was between 20 and 30 MΩ. Measurements were done on muscle fibers located in the deep part of the trunk accessible to microelectrodes from the interior of the fish after having removed the internal organs and a thin film lining the interior of the thorax. All the fibers are fast twitch type in this trunk region. Three different fibers were impaled with the same microelectrode that was slightly displaced laterally between each impalement. The mean resting membrane potential was found to be -78 ± 0.8 mV in 124 fibers from 4 fish.

3. I assume these are fast twitch fibers. If so, do they still twitch and contract when Nav1.4 channels are inactive/non-functional? Do the authors know if twitching occurs in mutants?

All the single fibers studied in the manuscript are indeed fast twitch fibers since the most peripheric muscle layer (slow muscle fibers) is almost entirely removed with the skin before collagenase treatment. The presence of NaV current, which is the characteristic of fast fibers in zebrafish, is also systematically tested by measuring a fast voltage-gated inward current in response to depolarization from a holding potential of -100 mV (see also Idoux et al., 2022, 2023).

To answer the reviewer question, we performed another series of experiments showing that WT fast muscle fibers contract in a form of a twitch but do not fire AP in response to direct electrical field stimulation. Indeed, new Fig. S2 below shows that stimulation pulses of high voltage on isolated fibers loaded with indo-1 AM did induce Ca^{2+} transients but that increased in size with stimulation voltage, giving evidence that the Ca^{2+} transients are not induced by an all-or-none AP but result from local electrotonic responses.

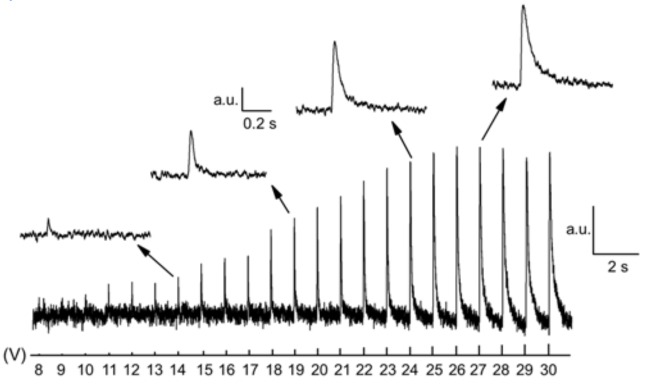

Figure S2: Effect of increasing voltage on Ca^{2+} signals elicited by field stimulation in an isolated WT zebrafish fast skeletal muscle fiber. Fibers were loaded with the acetoxymethyl ester form of the Ca^{2+} fluorophore indo-1 (2 µM; Invitrogen) during 30 minutes and then subsequently washed (30 minutes) in Tyrode solution to allow de-esterification of the dye. Indo-1 fluorescence was measured on an inverted Nikon Diaphot epifluorescence microscope equipped with a commercial optical system, allowing the detection of fluorescence at 485 nm by a photomultiplier (IonOptix, Milton, MA, USA) upon 360 nm excitation. Fluorescence signals were acquired at a sampling frequency of 10 kHz. Fibers were electrically stimulated at 1 Hz with voltage pulses of 0.5 ms duration through parallel platinum field electrodes. The voltage was gradually increased from 8 to 30 V, as indicated by the numbers below the lower trace. Arrows point to Ca^{2+} signals presented on an expanded scale. The stimulation frequency was 1 Hz and pulses duration was 0.5 ms. Graded responses to increasing voltages were observed in all the 8 tested fibers from 2 WT fish.

In line with this, Ca^{2+} transients evoked by high voltage stimulation were found to be not suppressed by exposing the fibers to a Tyrode solution containing 2 µM TTX (new Fig. S3 below). These data demonstrate that WT zebrafish fibers do not fire AP to produce Ca^{2+} transients and to contract in response to field stimulation.

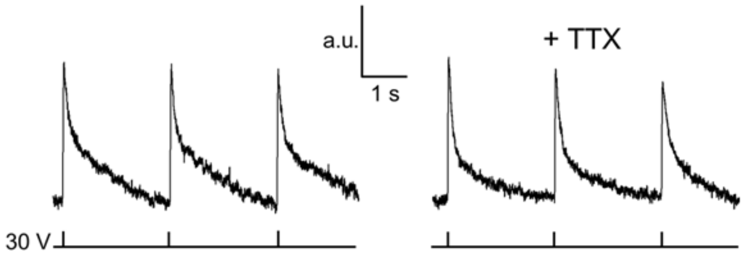

Figure S3: Absence of effect of TTX (2 µM) on Ca^{2+} signals evoked by field stimulation in an isolated WT zebrafish fast skeletal muscle fiber. Changes in indo-1 AM fluorescence was measured using the same procedures as the ones indicated in the legend of figure S2 in the absence (left) and after addition (right) of 2 µM TTX in the same fiber. Fibers were electrically stimulated at 0.4 Hz with pulses of 30 V amplitude and 0.5 ms duration. Absence of effect of TTX was observed in all the 10 fibers from 3 WT fish tested with this protocol.

The new figure S4 below shows that, in the same experimental conditions, Ca^{2+} signals in isolated mouse muscle fibers could be elicited by stimulation of much lower voltage and that these Ca^{2+} signals were completely and reversibly suppressed by applying 2 µM TTX.

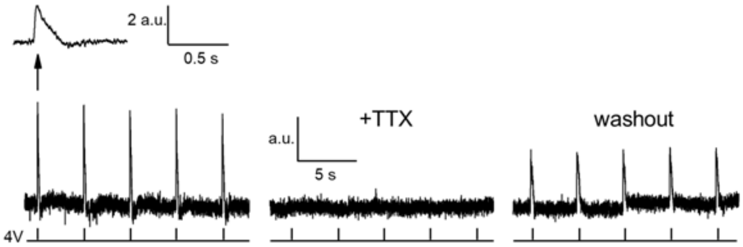

Figure S4: Effect of TTX (2 µM) on Ca^{2+} signals evoked by field stimulation in an isolated mouse skeletal muscle fiber. Changes in indo-1 AM fluorescence was measured using the same procedures as the ones indicated in the legend of figure S2 in the absence (left), in the presence (middle) and after washout (right) of 2 µM TTX in the same fiber. The stimulation frequency was 0.25 Hz and pulses duration was 0.5 ms. Note that all or none Ca^{2+} signals were evoked in response to voltage pulses of lower amplitude (4 V) as compared to zebrafish. The arrow points to Ca^{2+} signal presented on an expanded scale. An inhibitory effect of TTX was observed in all the 10 tested fibers from 2 mice.

Considering that NaV is absent in mutant fish and that even WT fibers do not fire AP (see point 4), we believe that it is worthless to test whether fish mutant fibers fire AP.
We propose to add these data in the new version of the manuscript, as new figure S2, S3 and S4. Indeed, demonstrating that fast single muscle fibers of WT zebrafish twitch without firing AP in response to field stimulation in contrast to mouse fibers in the same experimental conditions is a sound evidence that firing of AP is likely not required to produce contraction in zebrafish fast muscle fibers.

4. I am surprised that the authors did not attempt to record APs from mutants to show physiologically that a) these are indeed the only NaV channels that are present and functional, and b) that their mutations did indeed result in non-functional channels. I feel that these should be done.
We agree with the reviewer that such an information was lacking. We have performed voltage-clamp experiments on isolated fibers from double mutant zebrafish. As shown in new Fig. 4A below, depolarizing pulses of increasing amplitudes elicit only voltage-gated outward currents that were inhibited by TEA and replacement of Cl^- by $MeSO_3^-$ in all the 27 fibers from 7 mutant fish tested. This critical finding demonstrates that NaV1.4 is the only channel supporting voltage-gated inward currents and confirms that the two mutations result in non-functional NaV in muscle fibers of double mutants. These data have been added to the new version of the manuscript as new figure 4A.

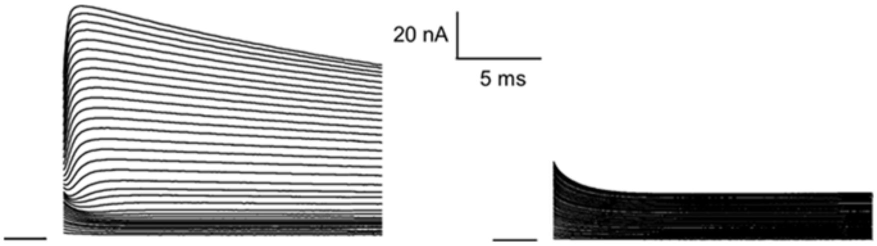

Figure 4A: Absence of voltage-gated inward currents in isolated fast muscle skeletal muscle fibers of the double mutant zebrafish. Left panel shows currents evoked by depolarizing pulses of increasing amplitudes (steps of 5 mV) from a holding potential of -110 mV applied from -100 to +40 mV in the presence of an external Tyrode solution. Right panel shows the effect of an external solution containing 20 mM TEA and in which external Cl- has been replaced by $MeSO_3$ on currents elicited by the same voltage protocol in the same fiber.

5. Discussion, Pg 11: The sentence in lines 8-13 contains repetitive text and should be reviewed.
The repetitive sentence “a single non-overshooting action potential followed by summated endplate potentials” has been removed.
